# Model-Based Electroencephalography Phenotyping Uncovers Distinct Neurocomputational Mechanisms Underlying Learning Impairments Across Psychopathologies

**DOI:** 10.1016/j.bpsgos.2025.100660

**Published:** 2025-11-29

**Authors:** Nadja R. Ging-Jehli, Rachel Rac-Lubashevsky, Krishn Bera, Megan A. Boudewyn, Cameron S. Carter, Molly A. Erickson, James M. Gold, Steven J. Luck, J. Daniel Ragland, Andrew P. Yonelinas, Angus W. MacDonald, Deanna M. Barch, Michael J. Frank

**Affiliations:** a Carney Institute for Brain Science, Department of Cognitive and Psychological Sciences, Brown University, Providence, Rhode Island; b Department of Psychology, University of California, Santa Cruz, California; c Department of Psychology, University of California, Irvine, California; d Department of Psychiatry and Behavioral Neuroscience, University of Chicago, Chicago, Illinois; e Department of Psychiatry, University of Maryland School of Medicine, Baltimore, Maryland; f Department of Psychology, University of California, Davis, California; g Department of Psychology, University of Minnesota, Minneapolis, Minnesota; h Department of Psychology, Washington University in St. Louis, St. Louis, Missouri

**Keywords:** Bipolar, Computational phenotyping, Depression, Reinforcement learning, Schizophrenia, Working memory

## Abstract

**Background:**

Major depressive disorder (MDD), bipolar disorder (BP), and schizophrenia (SCZ) involve learning impairments with poorly understood mechanisms. Understanding both the similarities and differences in these mechanisms is important to guide the development of new, targeted interventions.

**Methods:**

A total of 255 participants diagnosed with MDD (*n* = 54), BP (*n* = 47), or SCZ (*n* = 67) or without any clinical diagnoses (control [CTRL]) (*n* = 87) performed an associative learning task. Computational modeling quantified the mechanistic interplay between working memory (WM) and reinforcement learning (RL). The latent RL and WM signatures in the electroencephalography (EEG) dynamics showed shared and distinct neurocognitive mechanisms underlying learning.

**Results:**

All clinical groups showed learning impairments at the behavioral level. Model-based EEG analyses linked these impairments to distinct patterns in the dynamic interplay between latent RL and WM mechanisms, contrasting with the typical patterns observed in the CTRL group. SCZ was characterized by reduced neural markers of WM, weakening the cooperative influence of WM onto RL (reduced WM recruitment), and reduced integration of negative feedback. Conversely, MDD was characterized by reduced reciprocal influence of RL onto WM, reducing the tendency to upregulate WM contribution with reward history (impaired WM management). Finally, BP was characterized by deficits in both WM and RL recruitment, along with higher WM decay.

**Conclusions:**

Behavioral learning impairments that seem similar across clinical groups can be linked to distinct neurocognitive mechanisms via integrative neurocomputational modeling. Our approach provides insights into the interplay of underlying learning mechanisms and how they manifest differently across psychopathologies.

## Plain Language Summary

People with depression, bipolar disorder, and schizophrenia often show learning difficulties but the underlying causes may differ. By combining brain activity recordings with computational models, we identified distinct cognitive mechanisms driving these impairments. Our findings show how modeling and physiology can give insights into hidden decision dynamics behind learning difficulties. We also outline key steps needed to advance computational psychiatry tools toward clinical applications, including their potential use in guiding personalized treatment.

Learning impairments are prevalent across mental health conditions, yet their underlying mechanisms remain inconclusive (1,2). Computational psychiatry offers approaches to dissect latent mechanisms underlying neurocognitive and motivational processes that relate to learning and how their interplay reinforces distinct symptom patterns across psychopathologies (3, 4, 5, 6). This lays the foundation for predicting when and which mechanisms should be targeted in interventions.

Toward this endeavor, it is valuable to first evaluate whether mechanistic models can effectively differentiate transdiagnostic features that may seem identical at the behavioral level. Past research emphasizes the importance of complementing computational models with experimental paradigms that are sensitive to differentiate between model-simulated mechanisms and grounding them both in neuroscientific theory (3,7). Here, we demonstrate how such a mechanistic approach separates latent neurocognitive mechanisms of learning impairments. By doing so, we identified shared and distinct characteristics across major depressive disorder (MDD), bipolar disorder (BP), and schizophrenia (SCZ).

We focus on instrumental learning, which refers to the formation of stimulus-response associations via operant conditioning and relies on multiple neurocomputational mechanisms modulated by dopaminergic dynamics in distinct prefrontal–basal ganglia pathways (8, 9, 10). These mechanisms can be studied through neurocognitive frameworks of reinforcement learning (RL) and working memory (WM) (11,12). Combining RL and WM frameworks into the same model acknowledges that effective associative learning requires both slowly and robustly integrating the consequences of past actions (RL) and flexibly maintaining information (i.e., stimulus-action-outcome associations) over short periods (in WM). The RL-WM task has been shown to differentially probe these systems, producing behavioral learning curves consistent with the predictions of the mechanistic model in multiple (mostly nonclinical) studies (8,12, 13, 14, 15).

Here, we identified the unique neurocognitive learning dynamics among individuals with MDD, BP, and SCZ and those without clinical diagnoses (control [CTRL] participants) for the first time (Table 1). Combining the mechanistic RL-WM task (8) with model-based electroencephalography (EEG) analyses (12,13) allowed for validating the latent neurocognitive dynamics predicted by the computational model, tracing individual differences in real-time learning dynamics, and assigning cognitive interpretation to the extracted physiological measures. Although clinical groups exhibited similar behavioral impairments, we found that the model-based EEG analyses linked these impairments to alterations in different neurocomputational components.

**Table 1:** Background Characteristics of the Groups

	Groups			
	CTRL	MDD	BP	SCZ
Age, Years	34 (10)	32 (9)	37 (11)	36 (11)
Gender, Female:Male:Other	46:40:1	27:23:4	27:19:1	19:40:6
Race				
American Indian	1	1	2	2
Asian	8	1	1	1
Black/African American	23	11	10	26
White	48	37	34	33
School Years of the Participant	16 (2)	16 (2)	16 (3)	14 (3)
School Years of the Mother	14 (4)	14 (3)	14 (3)	15 (3)
School Years of the Father	14 (4)	15 (3)	15 (4)	15 (3)
Medication Status				
Antipsychotic	0	3	21	51
Mood stabilizer	0	3	23	12
Antidepressant	1	37	25	26
Antianxiety	3	14	13	13
Standardized IQ Scores Derived From WTAR-FSIQ	113 (12)	112 (12)	112 (11)	104 (16)

Values are presented as mean (SD) or *n*. A one-way ANOVA was conducted to test for age differences across groups (CTRL, MDD, BP, SCZ). The analysis revealed no significant differences in mean age among the groups (*F*_3,249_ = 2.26, *p* = .08). A χ^2^ test showed a significant association between gender and diagnostic group (χ^2^_3_ = 9.57, *p* = .022) (note that the “other” category was excluded from the analysis due to low counts in each group). However, post hoc pairwise comparisons of gender proportions across groups (adjusted using the Holm method) did not reveal any significant pairwise differences. Standardized residuals indicated that no individual cell deviated substantially from expected counts (all |*z*| <2), suggesting that the observed association may be modest and diffuse across groups. A χ^2^ test of independence revealed no significant association between race and diagnostic group (χ^2^_6_ = 11.40, *p* = .070). For the statistical analysis, individuals identifying as Asian or American Indian were combined into the “other” category due to low frequencies in each group. A one-way ANOVA was conducted to test for differences in school years across groups (CTRL, MDD, BP, SCZ), separately for participants, mothers, and fathers. The analyses revealed no significant group differences in maternal years of schooling (*F*_3,243_ = 0.12, *p* = .95) or paternal years of schooling (*F*_3,228_ = 2.39, *p* = .07). However, the one-way ANOVA for participants’ years of schooling suggested a statistically significant difference (*F*_3,248_ = 12.54, *p* < .001). Post hoc comparisons using Tukey’s HSD test showed that the SCZ group had significantly fewer years of schooling than each of the other groups (CTRL, MDD, BP; all *p*s < .001). χ^2^ tests of independence showed significant associations between diagnostic groups and medication status: antipsychotics (χ^2^_3_ = 128.31, *p* < .001), mood stabilizers (χ^2^_3_ = 65.98, *p* < .001), antidepressants (χ^2^_3_ = 80.05, *p* < .001), and antianxiety (χ^2^_3_ = 20.37, *p* < .001). Cognitively typical scores on the WTAR range on the standard FSIQ scores around a mean of 100 with SD of ±15. This shows that all the groups were in this normed range. Moreover, a one-way ANOVA for participants’ standardized IQ scores derived from WTAR suggested a statistically significant difference (*F*_3,249_ = 5.13, *p* = .002). Post hoc comparisons using Tukey’s HSD test showed that the SCZ group had significantly lower WTAR score than each of the other groups (CTRL, MDD, BP; all *p*s < .02).

ANOVA, analysis of variance; BP, bipolar disorder; CTRL, control; FSIQ, Full Scale IQ; HSD, honestly significant difference; MDD, major depressive disorder; SCZ, schizophrenia; WTAR, Wechsler Test of Adult Reading.

**Figure 1:**
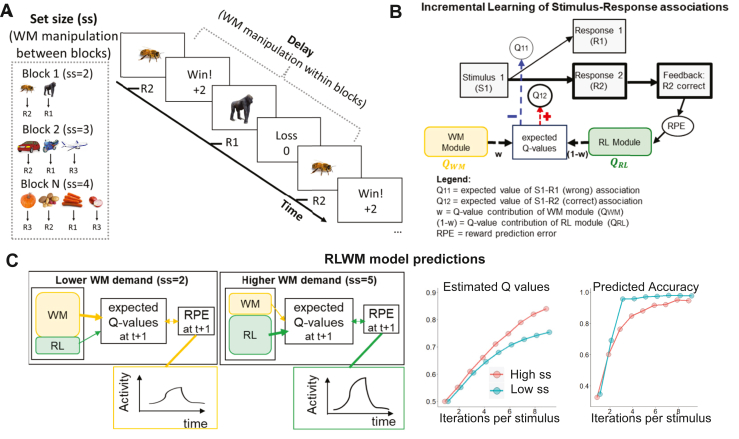
Characteristics of the reinforcement learning (RL)–working memory (WM) task and its computational model. **(A)** Participants learn associations between stimuli and 3 response options via feedback. Shown is the procedure of one task trial. WM load is parametrically manipulated with between-block manipulation of set size (ss) and within-block manipulation of delay. **(B)** The mechanistic computational model simulates the dynamic interaction of latent WM and RL components that contribute to the *Q* value–based learning process across trials. RPEs used for updating RL *Q* values are informed by RL expectations *Q*_RL_ but also cooperative impact of WM expectations (*Q*_WM_), weighted by the individual’s reliance on WM. Selected model parameters are summarized in the pink box. **(C)** Selected key model predictions, emulating how both WM and RL processes contribute to expected *Q* values and RPEs. Under lower WM demand, expected *Q* values can be retained in working memory, which diminishes RPEs in early learning stages. Conversely, under higher WM demand, working memory is above capacity, increasing the relative contribution of RL-based computations and leading to higher RPEs and RL *Q* values, despite worse accuracy. The predicted cooperative effect of RL and WM processes on estimated *Q* values and accuracy are displayed in the bottom subplots, respectively. These subplots are recreated from past studies (12,13).

In the RL-WM task, participants learn stimulus-response associations through trial and error (8,12,14, 15, 16) (Figure 1A). The task is structured into multiple blocks of trials, parametrically manipulating WM demand. In particular, WM demand varies between blocks due to differences in set size (the number of presented stimulus-response associations) and within blocks due to delay (the number of intervening trials before the same stimulus-response association is repeated). RL contributions are indexed by incremental increases in accuracy over time as a function of correctly repeated responses to a given stimulus-response association (reward history). Further details are presented in Methods and Materials and the Supplement.

The RL-WM model is fitted to trial-by-trial choice and includes a faster (but capacity-limited) WM module (mediated by active maintenance of stimulus-action-outcome contingencies within the prefrontal cortex [PFC]) (17, 18, 19, 20) and a slower (but capacity-free) RL module. Each module contributes to the formation of expected *Q* values for stimulus-response associations, of which the response with the highest *Q* value is selected (Figure 1B and Table 2). Capacity constraints make the WM module vulnerable to load and interference effects. Conversely, the slower RL module, thought to rely on corticostriatal synaptic plasticity, gradually strengthens learned associations through trial-by-trial feedback-driven reward prediction error (RPE). Hence, the expected reward value (*Q* value) of the chosen stimulus-response association increases progressively with accumulated reward history, easing the computational load initially handled by the WM module.

**Table 2:** Equations Specifying the RL-WM Computational Modeling Approach

$Q_{RL}{\left(s,a\right)}_{t}=\left\{ \begin{array}{c} Q_{RL}{\left(s,a\right)}_{t-1}+\alpha \cdot \left(1-Q_{RL}{\left(s,a\right)}_{t-1}\right),{if\;r}_{t-1}=1\\ Q_{RL}{\left(s,a\right)}_{t-1}+\alpha \cdot \gamma \cdot \left(-Q_{RL}{\left(s,a\right)}_{t-1}\right),{if\;r}_{t-1}=0 \end{array}\right.$	(1)
$Q_{WM}{\left(s,a\right)}_{t}=\left\{ \begin{array}{c} 1,{if\;r}_{t-1}=1\\ Q_{WM}{\left(s,a\right)}_{t-1}+\gamma \cdot \left(-Q_{WM}{\left(s,a\right)}_{t-1}\right),{if\;r}_{t-1}=0 \end{array}\right.$	(2)
$Q_{WM}{\left(s,a\right)}_{t}=Q_{WM}{\left(s,a\right)}_{t-1}+\phi \cdot \left(\frac{1}{n_{a}}-Q_{WM}{\left(s,a\right)}_{t-1}\right)$	(3)
$p\left(a|s\right)=\frac{\exp\left(\beta \cdot Q\left(s,a\right)\right)}{\sum_{i}\exp\left(\beta \cdot Q\left(s,a_{i}\right)\right)}$	(4)
$\omega =\rho \cdot \min\left(1,\frac{C}{ssz}\right)$	(5)
$pol_{mix}=\left(1-\omega \right)\cdot pol_{RL}+\omega \cdot pol_{WM}$	(6)
$pol_{final}=\left(1-\epsilon \right)\cdot pol_{mix}+\epsilon \cdot \left(\frac{1}{n_{a}}\right)$	(7)

Parameters are detailed in Modeling in Methods and Materials.

RL, reinforcement learning; WM, working memory.

Recent studies have shown that WM and RL systems also interact cooperatively (the RPEs needed for learning by the RL system are influenced by expectations that are held in WM) (10,12, 13, 14). For example, assume participants respond correctly and get positive feedback. When stimulus-outcome associations can be held in WM (during low load), this correct outcome will match WM-based expectations, reducing the RPE experienced by the RL module (Figure 1C).

To extract neural signatures of WM and RL components and how they interact, we used an established model-based EEG approach and focused on correct responses (see Methods and Materials) (12,13). Fitting the RL-WM model to behavioral data provided trial-by-trial measures of latent RL-based computations including *Q* values and RPEs. These model variables, together with WM manipulations (set size and delay), subsequently served as blueprints to extract trialwise neural RL and WM markers. The obtained markers consist of spatiotemporal EEG patterns quantifying the degree to which participants engage in WM and RL computations at any given time and brain (surface) location (12,13). The neural RL marker reflects brain correlates of the stimulus-response expectation within the RL system, providing a neural learning curve across stimulus iterations. Conversely, the neural RPE marker reflects individuals’ surprise after successful outcomes, which declines over trials as associations are learned.

To preview the main findings, all clinical groups showed poorer performance as compared with the CTRL group. First, BP and MDD were both characterized by poorer learning under higher than lower WM demand (i.e., higher set size effects). The model-based EEG analyses attributed these observable learning impairments to distinct underlying mechanisms. While BP was characterized by higher WM decay, MDD was characterized by overreliance on WM especially when it would have been beneficial to rely on RL (i.e., reduced effect of reward history on WM recruitment). We refer to this pattern as a deficit in WM management because it reflects the disruption of an adaptive process putatively related to frontostriatal-WM gating (21). Conversely, SCZ was characterized by WM impairments already under lower demand and reduced influence of negative compared with positive feedback on learning, replicating earlier effects in this task (16).

## Methods and Materials

### Participants

This study is part of the Cognitive [Computational] Neuroscience Test Reliability and Clinical Applications for Serious Mental Illness (CNTRaCS) Consortium. All participants were recruited between January 2020 and June 2022 from local clinics and surrounding communities of 5 testing sites: the University of Maryland, the University of California Davis, the University of Minnesota, the University of Chicago, and Washington University in St. Louis. All recruiting methods and experimental procedures were approved by a central institutional review board at Washington University. Demographics by group are presented in Table 1.

Diagnostic status for all participant groups was established using the Structured Clinical Interview for the DSM-5 (SCID). All patients were clinically stable with no medication changes in the previous month or anticipated in the upcoming month. Additionally, participants with MDD met DSM-5 criteria for at least 2 depressive episodes, at least one of which occurred within the past 3 years. Participants with SCZ were diagnosed as having either SCZ or schizoaffective disorder based on SCID described below. The CTRL group had no current psychiatric diagnosis, were not taking psychiatric medications, and reported no family history of psychosis. All participants were ages 18 to 63 years, reported no history of neurologic injury, and were free from a substance use disorder in the 3 months prior to study enrollment. For additional details, see Supplement, section 1.

After data preprocessing (described in subsequent sections), the analyses for this study included 223 participants (CTRL = 71, SCZ = 59, BP = 45, MDD = 48). Thirty-two participants (CTRL = 16, SCZ = 8, MDD = 6, BP = 2) were excluded during data preprocessing procedures. In particular, 23 participants were removed from all the reported EEG analyses due to a high EEG artifact rate (>40% in one or more of the conditions in the stimulus or feedback-locked data). Nine participants were removed because they had difficulties performing the task.

### Task

The RL-WM task differentiates between RL and WM mechanisms by assessing the impact of distinct task manipulations on learning processes. Participants learn stimulus-response associations through trial and error and subsequent feedback in a learning phase that consists of multiple blocks of trials. Error responses are rewarded with 0 points, whereas correct responses are probabilistically rewarded with either 1 or 2 points (12,14). The task is structured into multiple blocks of trials, with systematic manipulations both between and within blocks to parametrically adjust WM demand. Set size refers to the number of stimulus-response associations to be learned per block. Delay refers to the number of intervening trials since a stimulus was last correctly paired with a response. For additional details, see Supplement, section 1.

### Modeling

Fitted to participants’ choice data, the RL-WM model characterizes learning via RL and WM processes (12,13) (Table 2). Both processes maintain a separate state-action value representation, which is updated using temporal-difference style updates. The learning rate of the WM system $\alpha_{\text{WM}}$ is fixed at 1 to capture fast WM updating. The WM capacity is denoted by *C*, and the decay is modulated by a decay parameter ϕ. The final action policy is derived from a weighted sum of RL and WM policies. The parameter ρ denotes the participant’s overall extent of reliance on the WM process (over the RL process).

The trialwise learning updates for the RL and WM processes are given by equations 1 and 2 in Table 2, where *s* refers to the current trial stimulus, *a* is the chosen action, $r_{t-1}$ is the reward obtained at the current trial, α is the RL learning rate, and γ is the perseveration parameter. The perseveration parameter captures the neglect of negative feedback.

#### WM Decay

The model simulates WM decay by gradually reducing the WM-based *Q* values toward their initial values of 1/*n*_*a*_, with *n*_*a*_ representing the number of possible actions. The decay rate is controlled by ϕ. In Table 2, equation 3 denotes WM decay at every trial.

#### Behavior Policy

For each trial, the *Q* values generated by the RL and WM processes were converted into choice action probabilities via a softmax function, shown in Table 2, equation 4, with a fixed inverse-temperature parameter, β. β was fixed to 100 across all participants to avoid significant tradeoffs with other model parameters as in past work (22). The derived policies, pol_RL_ and pol_WM_, were then integrated into a weighted mixture policy, pol_mix_, which depended on the (blockwise) degree of WM reliance, represented by ω. In Table 2, equation 5 shows blockwise computation of ω. The ρ parameter is the participant’s baseline tendency to rely on WM across contexts (blocks), the *C* parameter represents the participant’s WM capacity, and ss is the set size or the number of unique stimuli encountered in each block. In Table 2, equation 6 computes the mixture policy derived from pol_RL_ and pol_WM_.

#### Undirected Noise

The final choice policy for the training phase, pol_final_, is a weighted mixture of the model-derived choice policy (craven by RL and WM contributions) and a uniform random policy, representing undirected noise driven by ε, which estimates the probability of making a random choice, independent of learned or remembered values. In Table 2, equation 7 formalizes this policy mathematically. Conceptually, ε captures latent decision noise or attentional disengagement and is commonly interpreted as a lapse rate in computational modeling.

#### Model Fitting

The model was fit to participant-level data using a constrained maximum likelihood estimation (MLE) procedure. We used Nelder-Mead optimization as implemented in *scipy.optimize* (23). Parameter *C* was estimated as an integer value by looping MLE estimation over the integer parameter range (2 to 5) to get the best-fit combination of all parameters. Each estimate was obtained after running the optimization procedure with 40 different initializations (for each value of *C*) to increase the chance of finding the global optimum. All other parameters were constrained in the range (0 to 1) except for the α parameter, which was constrained to the range (0 to 0.3) to avoid identifiability issues when α ∼ α_WM_. The previous RL-WM modeling results (12,13,16,24) have already shown that α << α_WM_ (1) and α < 0.3. Note that, following previous conventions of the RL-WM task with the same model (12,13,25), α_WM_ is fixed at 1 to reflect the assumption that WM updates deterministically upon feedback and has perfect recall. To maintain identifiability between the RL and WM systems, α must be kept sufficiently lower than 1.

### Electroencephalography

We used a Brain Products active electrode ActiCHamp system with 29 scalp electrodes and 3 electro-ocular electrodes for recording electrophysiological activity. A detailed description of our preprocessing pipeline is provided in previous studies and in the Supplement, section 1 (26,27). Preprocessing of EEG data was done in MATLAB (version R2022b–R2024a; The MathWorks, Inc.) using the EEGLAB Toolbox (28) and the ERPLAB plugin (29). We highpass filtered the raw data (sampling rate, 500 Hz; antialiasing filter, 130 Hz) using a Butterworth filter (half-amplitude cutoff at 0.05 Hz with a 12 dB/octave slope) and screening for malfunctioning channels (i.e., channels with more than one-third of unusable data). We then used independent component analysis (ICA) to correct for eye blinks and horizontal eye movement artifacts. Channels identified as nonfunctional were excluded from the ICA and later interpolated using a spherical spline algorithm. Approximately 67% of participants had no malfunctioning channels requiring interpolation (CTRL, 72.5%; SCZ, 60.34%; BP, 59.5%; MDD, 74.5%), whereas the rest had between 1 and 6 channels interpolated (average for CTRL, 2.56; SCZ, 2.78; BP, 2.24; MDD, 2.54). The continuous EEG was downsampled to 125 Hz, then reduced to a selected window of −100 to +700 ms twice.

### Model-Based EEG Analyses

To extract the neural correlates in the EEG signal of conditions of interest, we used a mass univariate approach (13). A multiple regression analysis was conducted for each participant, in which the EEG amplitude at each electrode site and time point was predicted by the conditions of interest: set size (number of stimulus-response-outcome associations given in a block), model-derived RL expected value (denoted as *Q*), delay (number of trials since the same stimulus was presented and a correct response was given), and the interaction of these 3 regressors, while controlling for other factors such as reaction time (log-transformed) and trial number within block. To account for the remaining noise in the EEG data, the EEG signal (at each time point and electrode) was *z* scored across all trials and so were all the predictors before they were entered into the robust multilinear regression analysis (12,13). The neural RL marker (of expected *Q* values) and neural RPE marker were subsequently used as predictors in general mixed-effect regression analyses. The neural RL marker was calculated by averaging the values of the final 4 iterations of each stimulus to capture asymptotic performance. This was done for each stimulus-response association within each participant. For additional details, see Supplement, section 1.

## Results

A total of 255 participants—CTRL (*n* = 87) or those with SCZ (*n* = 67), MDD (*n* = 54), or BP (*n* = 47)—performed the RL-WM task during EEG recording (Table 1). We first summarize findings from analyses including all participants, highlighting the dissociable computations related to WM and RL in both behavioral and EEG measures and replicating past results (12, 13, 14). This builds the foundation for interpreting clinical similarities and differences in the second part. To dissociate WM and RL effects on performance measures, we used mixed-effect regressions whose complete outputs are provided in the Supplement.

### General Findings

**Figure 2:**
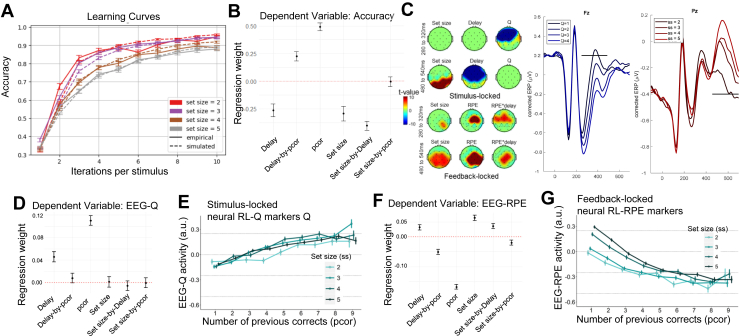
Analyses across all participants replicate previous reinforcement learning (RL)–working memory (WM) behavioral, electroencephalography (EEG), and mechanistic modeling findings. **(A)** Accuracy (averaged across trials and participants) curves by number of stimulus iterations show continuous learning over time that varies with set size. Vertical bars represent within-subject SEs. **(B)** Coefficient weights from logistic mixed-effect regressions with trial-based accuracy as the dependent variable and covariates: set size, delay, reward history (pcor), and their interactions. This shows dissociable contributions of WM components (delay, set size, and their interactions) and incremental RL (effect of previous corrects). See Table S1 for regression output. **(C)** EEG-based markers of WM and RL during decision making and feedback processing. The topographical scalp maps illustrate the effects of each predictor (set size, delay, *Q*) across an early (top row) and later (bottom row) time window. The maps are presented for stimulus-locked events (left plots) and for feedback-locked events (right plots). These spatiotemporal patterns induced by generalized linear model (GLM) can also be visualized by classical event-related potentials (ERPs) correcting for other terms in the GLM. Here, corrected ERP plots show the effect of the 2 main predictors discussed in Results (see Figure S1 for analogous plots of all predictors), namely: RL markers in blue (by RL value quartiles) and neural set size markers in red (by set size) on the voltage of significant electrodes (Fz and Pz both at central lines). Horizontal black lines reflect significant time points after permutation correction. **(D)** Coefficient weights from mixed-effect regressions with neural RL markers as the dependent variable and predictors: set size, delay, reward history (pcor), and their interactions. As expected, pcor and delay contributed to neural RL markers consistent with past studies, showing more RL recruitment under longer delay (12,13). See Table S2 for regression output. **(E)** Neural RL markers, indexing incremental RL, increased with reward history (pcor). Vertical bars represent within-subject SEs. Indices were extracted from trial-by-trial model-based EEG analyses using trial-based *Q* value estimates as a blueprint. **(F)** Coefficient weights from regressions with neural reward prediction error (RPE) markers as the dependent variable and predictors: set size, delay, reward history (pcor), and their interactions. As expected, neural RPE was inversely related to pcor but positively related to set size. See Table S3 for regression output. **(G)** Neural RPE markers decreased with reward history (pcor). Vertical bars represent within-subject SEs. Indices were extracted from model-based EEG analyses using trial-based RPE estimates as a blueprint.

Participants learned the stimulus-response associations (β = 0.78, SE = 0.04, *p* < .001) (Figure 2A). Figure 2A also shows that the RL-WM model (introduced earlier) captured these learning curves well.

#### Instrumental Learning Mitigates the Negative Performance Effects of Higher WM Demand

We manipulated WM demand by varying set size (number of stimulus-response associations) and delay (number of trials between successive presentations of the same stimulus). As expected, trial-by-trial accuracy significantly decreased under higher WM demand (Figure 2B). However, these detrimental effects diminished with accumulated reward history (10,12, 13, 14,16).

#### Model-Based Neural Markers Dissect WM and RL Contributions

The model-based EEG analyses used the computational parameters to extract neural markers of RL and WM components. By identifying clusters of neural activity associated with these components (see Methods and Materials), we tracked the dynamic evolution of these latent RL and WM components at a neural level over time. The spatial distribution of these neurocomputational markers within the temporal windows where their association was significant. The neural RL marker of *Q* values (subsequently referred to as neural RL markers) produced early frontal activity during decision making, while the neural WM markers showed later parietal (for set size) and frontal (for delay) activities. Hence, RL components contributed to earlier learning, whereas WM components contributed to later stages (16).

#### Neural RL Markers

As expected, greater neural RL markers were related to higher accuracy during the asymptotic phase, as indicated by a significant interaction between RL marker and reward history (β = 0.045, SE = 0.014, *p* = .002). The corresponding regression weights are presented in Figure 2D. As successful experiences accumulated (reward history), participants transitioned from WM to RL computations, consistent with RL-WM model predictions (8,10,12, 13, 14,16) (Figure 2E).

To separate WM and RL contributions, we estimated a linear mixed-effects regression with neural RL markers as the dependent variable and predictors including behavioral and neural WM components (set size, neural set size markers), RL components (reward history), and their interactions (Table S4). This revealed positive main effects of both reward history (β = 0.012, SE = 0.007, *p* < .001) and set size (β = 0.052, SE = 0.006, *p* < .001), implying increased RL engagement not only under asymptotic learning but also under higher WM demand. We also found a negative main effect of neural set size marker (β = −0.33, SE = 0.007, *p* < .001), suggesting that neural markers of WM are indicative of reduced RL engagement over and above the manipulated WM demand (i.e., objective set size). Finally, we found a significant interaction between reward history and neural set size marker (β = 0.011, SE = 0.005, *p* = .021). As reward history accumulated, greater neural set size markers were related to enhanced RL computations, which are especially beneficial under higher WM demand (i.e., higher set sizes) as detailed in the next paragraphs (12,13). We will later demonstrate that MDD specifically is associated with impairments in this interactive RL-WM mechanism.

#### Neural Set Size Markers

Theoretical models of frontostriatal circuits suggest that WM influences instrumental learning, and RL also reciprocally influences gating of information into and out of WM, supporting adaptive management (21,30). Accordingly, we found that higher neural set size markers reflected not only WM challenges (due to their positive associations with WM demand as indexed by set size) but also efficiency in WM management (due to their positive associations with RL processes as indexed by reward history). This is evidenced by a linear mixed-effects regression (Table S5), showing that neural set size markers increased with both set size (β = 0.117, SE = 0.004, *p* < .001) and reward history (β = 0.023, SE = 0.004, *p* < .001), along with a significant interaction between them (β = 0.011, SE = 0.004, *p* = .015). As participants accumulate reward history, they form stronger stimulus-response associations within RL, freeing the WM system to efficiently prioritize how to manage its resources under larger set sizes.

We next examined whether neural set size markers predicted accuracy, beyond set size and reward history effects (Table S6). Indeed, higher neural set size marker predicted higher accuracy under higher WM demand as indicated by a positive interaction between neural set size marker and set size (β = 0.043, SE = 0.016, *p* = .009). This association intensified with reward history, as indicated by the positive interaction between neural set size marker and reward history (β = 0.026, SE = 0.013, *p* = .035), suggesting a positive influence of RL on WM management.

#### Neural RPE Markers

A core tenet of RL is that RPEs are computed as a difference between experienced and expected outcome, and hence, neural markers of RPE should be larger if preceded in that same trial by lower neural RL markers (i.e., lower expectations). Indeed, higher neural RPE markers were associated with lower preceding neural RL markers (β = −0.03, SE = 0.01, *p* < .001), representing reduced surprise during outcomes, and consistent with past findings (31). This effect held even when including the RPE from the RL-WM model as an additional predictor, demonstrating the neural RL markers reflecting subjective trialwise expectations over and above what could be gleaned from the fitted RPEs based on behavior only (Table S7).

As expected, neural RPE markers also evolved across trials, decreasing with reward history but increasing under higher WM demand (higher set size and/or longer delays) (Figure 2F). Hence, neural RPEs declined as learned associations manifested, but remained more pronounced under higher WM demand (Figure 2G) consistent with the cooperative RL-WM model and past findings (10,12,13). In particular, we estimated a linear mixed-effect regression with neural RPE markers as the dependent variable and predictors including set size, neural set size marker, and their interaction (Table S8). This showed that neural RPE markers were pronounced under higher set size (β = 0.066, SE = 0.004, *p* < .001), highlighting the expected cooperative contribution of WM for RL-driven learning (12).

### Clinical Findings

**Figure 3:**
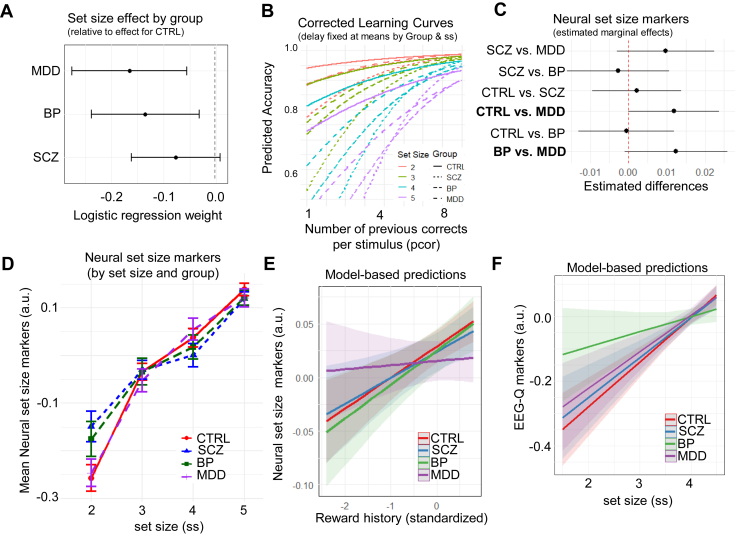
Major depressive disorder (MDD) and bipolar disorder (BP) show similar disruptions with increasing set size but are related to distinct model-based neural markers. **(A)** Logistic regression with accuracy as the dependent variable and predictors including delay, set size, reward history, group, and their interactions. Dots represent point estimates, and lines represent 95% CI. This analysis showed larger set size effects for the MDD and BP groups compared with the control (CTRL) group. See Table S10 for regression output and Figure S4 for delay and reward history effects. **(B)** Graphical illustration of the pronounced set size effect specific to MDD, BP, and schizophrenia (SCZ) with corrected learning curves by fixing delay at mean level within each set size and group. We also estimated a regression with accuracy as the dependent variable and predictors including group, set size, delay, reward history, and their interactions. Compared with the CTRL group, the BP and MDD groups had more trouble maintaining accuracy as set size increased across blocks. See Figure S5 for plots by group. **(C)** Pairwise group differences in coefficient weights from linear mixed-effect regression with neural set size marker as the dependent variable and group as covariate. Dots represent point estimates, and lines represent 95% CI. See Figure S6 for group comparison across all neural markers. **(D)** Neural set size markers (arbitrary units [a.u.]) show parametric increases with set size (indexing higher working memory [WM] demand), with weaker increases in BP and SCZ. Group means were calculated by first averaging within group and set size and then by group. Dots represent means, and vertical bars represent SEMs. **(E)** Model-based predictive plot of reward history on neural set size markers (arbitrary units) by group. Overall, participants showed increased engagement of neural indices of WM with reward history; this effect was blunted in MDD. The linear mixed-effect regression model included the neural set size marker as the dependent variable and predictors: set size, reward history, and group. See Table S11 for regression output. **(F)** Model-based predictive plot of set size effect on neural reinforcement learning (RL) marker (arbitrary units) by group. Neural RL markers served as the dependent variable, whereas group, WM components (set size, neural set size markers), reward history, and their interactions served as predictors. See Table S12 for regression output.

The group-specific learning curves, demonstrating learning in all groups, are presented in Figure S2. The SCZ and BP groups had significantly lower accuracy compared with the CTRL group (Figure S3), while the MDD and BP groups showed larger accuracy decrements as WM demand increased compared with the CTRL group (Figure 3A).

#### Similar WM Deficits in BP and MDD at the Behavioral Levels

Compared with the CTRL group, the BP and MDD groups had more trouble maintaining accuracy as set size increased across blocks (Figure 3A, B); see figure caption for regression model specification. The impact of set size on accuracy was moderated by the neural set size marker. Once neural set size was added as a predictor, the greater set size effects of MDD on accuracy were rendered insignificant, a moderation that was not observed for the CTRL or BP group (Table S9).

#### Distinct Neural Mechanisms Contributing to WM Deficits in BP and MDD

Although BP and MDD showed similar behavioral effects of set size, group comparisons in the model-based EEG markers suggested that these were due to distinct underlying mechanisms. The MDD group had significantly decreased neural set size markers compared with the BP and CTRL groups, which did not differ from each other (Figure 3C). Whether the reduced neural set size markers are due to less WM recruitment or deficits in WM management is not immediately evident. However, our computational analyses below suggest that reductions in neural set size markers in MDD were due to deficits in WM management. This is because MDD and CTRL groups displayed similar rises in neural set size markers with increased set size (Figure 3D), suggesting comparable WM activation in response to higher WM demand. Moreover, we showed in the previous section that increased neural set size markers were associated with improved performance.

#### MDD is Characterized by Inefficient WM Management

In part 1 of Results, we showed that neural set size markers increased with reward history, putatively reflecting RL processes that recruit WM. Figure 3E shows that this association was significantly weaker for those with MDD (β = −0.026, SE = 0.012, *p* = .003) compared with CTRL participants (β = 0.029, SE = 0.007, *p* < .001) who did not differ from those with SCZ (β = −0.003, SE = 0.012, *p* = .760) or BP (β = −0.002, SE = 0.012, *p* = .867) (Table S13). This suggests deficits in the support of RL (reward history) on WM management (indexed by neural set size markers). We did not find evidence for the alternative explanation of deficient WM recruitment in response to higher WM demand (Figure 3D). Increases in set size were related to larger neural set size markers in the CTRL group (β = 0.134, SE = 0.007, *p* < .001) with no significant difference in the MDD group (β = −0.007, SE = 0.012, *p* = .532). These findings distinguish the MDD group from BP and SCZ groups, who both showed deficits in WM recruitment.

#### BP and SCZ are Characterized by Reduced WM Recruitment

The BP and SCZ groups exhibited significantly smaller increases in neural set size markers under higher WM demand (increased set sizes) compared with MDD and CTRL groups (CTRL: β = 0.133, SE = 0.010, *p* < .001; MDD: β = −0.007, SE = 0.016, *p* = .661; SCZ: β = −0.039, SE = 0.016, *p* = .014; BP: β = −0.036, SE = 0.017, *p* = .037) (Table S14). This suggests potential deficits in WM recruitment, further supported by the observation that both groups experienced higher WM load at lower demands as indicated by their already elevated neural set size markers in blocks with lower set sizes (16) (Figure 3E).

#### BP is Characterized by Lower Neural RL Markers

**Figure 4:**
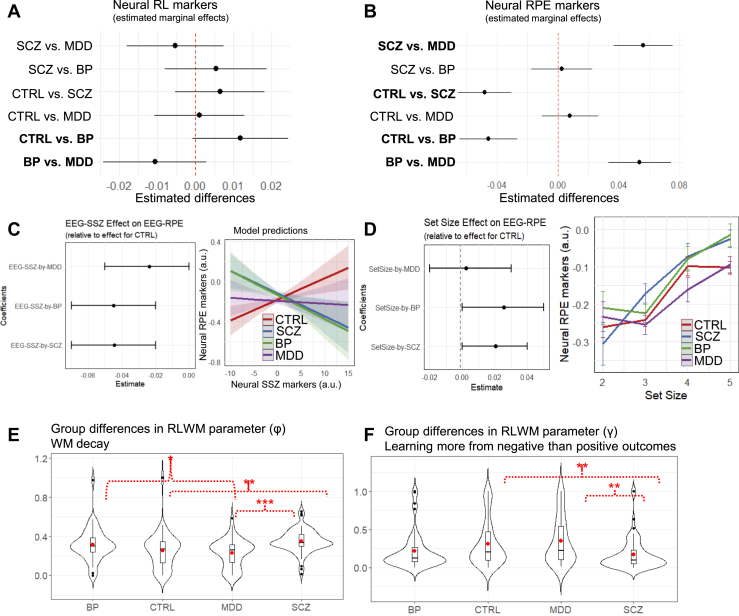
Bipolar disorder (BP) and schizophrenia (SCZ) show lower working memory (WM) contributions to reinforcement learning (RL) computations due to distinct underlying sources. **(A)** Pairwise group differences in coefficient weights from linear mixed-effect regression with neural RL marker (of *Q* values) as the dependent variable and group as covariate. Dots represent point estimates, and lines represent 95% CI. **(B)** Pairwise group differences in coefficient weights from linear mixed-effect regression with trial-based neural reward prediction error (RPE) marker as the dependent variable and group as covariate. Dots represent point estimates, and lines represent 95% CI. **(C)** Left plot: Coefficient from linear-mixed regression with neural RL-RPE marker as the dependent variable and neural set size marker, set size, and group as predictors demonstrates grouping of BP and SCZ in terms of a diminished effect of WM onto RL computation. Right plot: Plotting neural RPE markers as a function of neural set size markers from the regression-based model shows the predicted positive association in control (CTRL) participants (suggesting functional cooperative RL-WM interaction) but not in clinical groups. See Table S15 for regression output. **(D)** Left plot: Coefficient from linear-mixed regression with neural RL-RPE marker as the dependent variable and task variables and group as covariates demonstrates larger neural set size effects for the BP and SCZ groups compared with the CTRL group. Right plot: Plotting neural RPE markers across set size and by group shows greater activation for BP and SCZ under higher WM load (increased set size). Dots represent empirical data (averaged by participant and then by group). Vertical bars represent SEMs. See Table S16 for regression output. **(E)** Distribution of the RL-WM model parameter ϕ (WM decay) by group. Figure S7 provides group comparison across all parameters, and Figure S8 shows distributions after outlier removal. The one-way analysis of variance (ANOVA) suggested a statistically significant difference in model parameter between the groups (*F*_3,251_ = 8.020, *p* < .001). To identify which groups differed significantly, a Tukey’s honest significant difference (HSD) post hoc test was performed. Significant results of the Tukey’s HSD test were as follows: 1) mean major depressive disorder (MDD) minus mean BP = −0.087; 95% CI, −0.168 to −0.006; *p*_adjusted_ = .030; 2) mean SCZ minus mean CTRL = 0.096; 95% CI, 0.031–0.163; *p*_adjusted_ = .001; and 3) mean SCZ minus mean MDD = 0.123; 95% CI, 0.123–0.049; *p*_adjusted_ < .001. **(F)** Distribution of model parameter (γ) by group. This parameter indexes learning more from negative than positive outcomes and is estimated by applying the RL-WM computational model to accuracy as a task performance measure. Figure S7 provides group comparison across all parameters, and Figure S8 shows distributions after outlier removal. The one-way ANOVA suggested a statistically significant difference in model parameter between the groups (*F*_3,251_ = 6.058, *p* < .001). To identify which groups differed significantly, a Tukey’s HSD post hoc test was performed. Significant results of the Tukey’s HSD test were as follows: 1) mean SCZ minus mean CTRL = −0.144; 95% CI, −0.256 to −0.031; *p*_adjusted_ = .006; and 2) mean SCZ minus mean MDD = −0.178; 95% CI, −0.305 to −0.052; *p*_adjusted_ = .002.

Beyond the blunted WM recruitment, the BP group also showed significantly blunted neural RL markers compared with CTRL and MDD groups (Figure 4A). The tendency to recruit neural RL with increasing set size (when RL is more useful) was significantly more pronounced in the CTRL group (β = 0.069, SE = 0.011, *p* < .001) than in the BP group (β = −0.046, SE = 0.019, *p* = .015), as shown in Figure 3F. The SCZ and MDD groups did not differ from the CTRL group (SCZ: β = −0.007, SE = 0.017, *p* = .673; MDD: β = −0.012, SE = 0.018, *p* = .486). Moreover, the previously established negative association between neural set size markers and neural RL markers was significantly more pronounced in the BP group (β = −0.034, SE = 0.012, *p* = .004) and SCZ (β = −0.031, SE = 0.011, *p* = .004) as compared with the CTRL group (β = −0.314, SE = 0.007, *p* < .001). The MDD group did not differ significantly from the CTRL group (β = −0.002, SE = 0.011, *p* = .086) (Table S12). These findings indicate that BP was characterized by deficits in both WM and RL recruitment (particularly under higher WM demand). Conversely, SCZ displayed deficits primarily in WM recruitment (irrespective of WM demand), while MDD showed challenges in adaptive WM management rather than recruitment.

#### SCZ is Characterized by Weaker WM-RL Cooperation Under Higher WM Demand

If the WM and RL modules interact cooperatively, we would anticipate a positive relationship between neural markers of set size and RPEs (31) (Figure 1C). We replicate this pattern in the CTRL group (β = 0.020, SE = 0.007, *p* = .009) (32, 33, 34). However, Figure 4C shows that this cooperative effect was reduced for all the clinical groups compared with the CTRL group (SCZ: β = −0.050, SE = 0.011, *p* = .001; BP: β = −0.044, SE = 0.012, *p* = .005; MDD: β = −0.023, SE = 0.012, *p* = .049), and even more so for the SCZ group under higher objective WM demand indexed by increased set size (β = −0.029, SE = 0.012, *p* = .014) (Table S15). These results suggest disruptions in the typically (31,33) cooperative relationship between the RL and WM modules among the clinical groups. These effects were not simply due to blunted RPE signaling overall; indeed, when analyzed over all trials irrespective of neural WM markers, SCZ and BP groups exhibited significantly higher neural RPE markers compared with MDD and CTRL groups (Figure 4B). Moreover, the SCZ and BP groups had significantly higher increases in RPE under higher WM load, as shown in Figure 4D. Thus, our finding of reduced neural RPEs with increasing neural markers of WM is particularly indicative of a disruption in cooperative interactions between these brain systems, putatively reflecting reduced impact of the PFC on RL signaling (see Discussion).

#### Comparing Model Parameters

Correlational analyses between the neural set size marker (averaged by participant) and the participant-specific RL-WM parameters provided a better understanding of individual differences within each group (3). We complement the previous clinical findings with 3 additional insights.

First, those with BP and SCZ had significantly higher WM decay (ϕ) than those with MDD, which may explain why they showed deficits in WM recruitment and performance impairment already at lower WM demand as compared with those with MDD who showed deficits in WM management but not WM recruitment (Figure 4E). Focusing on individual differences with between-subject correlational analyses, those with more WM decay (higher ϕ) showed lower neural RL markers (*r* = −.424, *p* = .008). Given that lower neural RL markers were associated with lower accuracy, these results further support the previous conclusion that BP is characterized by deficits in both WM and RL recruitment.

Second, participants with MDD who relied more on WM than RL components (higher ρ) had lower neural set size markers (*r* = −0.293, *p* = .043). While greater reliance on WM might seem beneficial for performance, it can be detrimental especially when WM management is deficient, as observed in MDD. Indeed, those with higher WM reliance (ρ) performed worse under higher WM demand (increased set size; β = −7.77, SE = 0.92, *p* < .001) as shown in a regression with accuracy as the dependent variable and predictors including set size, ρ, and their interactions.

Third, we established earlier that SCZ differed from BP in that they exhibited deficits only in WM recruitment, while BP showed deficits in both WM and RL recruitment. The RL-WM parameter analyses revealed further that SCZ also demonstrated more perseveration, learning less from negative than positive outcomes (lower γ) (see Figure 4F).

In Figure S7, we compare the groups across all parameters. We did not find group-level differences in undirected decision noise (ε). This does not contradict previous findings of increased behavioral stochasticity in clinical populations (25,35) because our computational model includes multiple parameters (e.g., WM decay, learning rate, and WM-RL weighting) that can capture variability that is otherwise attributed to noise. This suggests that what appears as stochasticity in simpler models may reflect interpretable process-level dynamics when examined through a richer mechanistic lens.

To evaluate the robustness of group differences in RL-WM parameters, we conducted sensitivity analyses after removing outliers (detailed in the Supplement, section 1). These additional analyses led to the exclusion of 56 participants (CTRL = 19, BP = 15, MDD = 5, SCZ = 17) who showed extreme values in 1 or more parameters. Figure S8 shows that the overall pattern of group differences remained unchanged, with some effects appearing even stronger. We also refer to Figure S9 for additional recovery analyses of estimated model parameters.

## Discussion

This study is the first application of the RL-WM task to characterize cognitive mechanisms across multiple psychopathologies and with model-based EEG. Some groups showed similar behavioral impairments such as MDD and BP that exhibited greater accuracy decrements under higher WM demand than the SCZ group that showed impairments already under lower WM demand. The model-based EEG analyses linked these behavioral impairments to distinct deficits in neural components. Our findings demonstrate the power of integrative model-based approaches for the identification of distinct cognitive and neural signatures across groups. This is important because MDD, BP, and SCZ have been associated with learning impairments but the underlying mechanisms contributing to these impairments remained inconclusive thus far (32,36). Future studies should examine symptom dimensions and severity within and across diagnoses and, in larger samples, further dissect the mechanisms underlying learning impairments. This study serves as a foundational first step.

We also advance theories within the broader domain of computational cognitive neuroscience. Theoretical models of frontostriatal circuits have proposed not only that WM influences instrumental learning but also that RL reciprocally supports WM management (21). Our findings provide empirical support for these theories, demonstrating that the neural set size marker increased not only with subjective WM load under higher demand but also with reward history, suggesting improved WM management as RL processes alleviate the load on WM resources.

Recent nonclinical studies suggest that RL and WM processes are not independent modules. Rather, behavioral, functional magnetic resonance imaging, and EEG studies have suggested that WM influences RL (10,12, 13, 14). Thus, observations of diminished behavioral performance may partly reflect diminished influence of WM onto RL processes, which are posited to involve top-down prefrontal interactions with striatum. Indeed, previous studies suggest that SCZ are less responsive to top-down instructions that rely on working memory representations in the prefrontal cortex to guide striatal RL (31). This is important to consider given that past studies showed that behavioral impairments in RL tasks are more strongly tied to deficits in WM function, whereas RL-specific computations seem to be relatively intact in SCZ (14,33). Moreover, our finding of reduced neural WM markers in SCZ aligns with established endophenotypes involving deficits in attention, cognitive control, and abnormalities in sleep-related physiology, such as reduced sleep spindles and slow-wave activity (34,37, 38, 39). We view our approach as a bridge between mechanistic computational modeling and traditional endophenotypic frameworks: By estimating latent neurocognitive dynamics from behavior and EEG, future research can begin to map these task-derived markers onto broader biological traits, including sleep architecture and genetic risk profiles and their predictability of symptoms over time.

The interplay between WM and RL has been a key area of focus in cognitive studies, shedding light on how these systems collaboratively influence learning (13,15,40). Consistent with past findings, we found that higher experienced WM load (indexed by neural set size marker) increased the reliance on RL components by enhancing RPE signals (12,13). This aligns with neurocomputational models that emphasize dopamine’s role not only in RL mechanisms but also in modulating interactions with WM, affecting both learning and memory processes. Traditionally, dopamine has been primarily studied within the RL system, where phasic changes signal RPE in the striatum (41, 42, 43). Increasingly, studies are exploring how dopaminergic signaling related to RL also supports the manipulation and maintenance of WM content in the PFC (21,44,45).

Recent work has demonstrated the utility of EEG in identifying transdiagnostic learning impairments using event-related potential components such as the feedback-related negativity and P3 (46). Our study provides a complementary perspective focused on the mechanisms that give rise to neural dynamics. In particular, our model-based EEG analyses dissociate RL from WM and link their trial-by-trial latent dynamics to observed choices in a way that is mechanistically interpretable.

Examining how neurocognitive, motivational, and situational mechanisms interact at different timescales is crucial for building a more holistic understanding of symptom patterns across psychopathologies (3, 4, 5, 6). Such a mechanistic understanding forms the basis for identifying and predicting the mechanisms to be targeted in interventions. This study sets the groundwork by disentangling neurocomputational mechanisms contributing to clinical impairments within a specific context, namely, in an abstract associative learning task. While this serves as an important starting point, it is essential to recognize that clinical symptoms often manifest and vary across different contexts.

By establishing that shared learning impairments at a behavioral level can arise from different latent mechanisms in MDD, BP, and SCZ, we lay a rigorous foundation for future dimensional and individualized approaches. Such layered modeling, moving from diagnostic groupings to transdiagnostic and symptom-specific signatures, will be critical for advancing precision psychiatry. We acknowledge that group-level differences may be influenced by demographic variability. We deliberately attempted to match our clinical and control group variables such as age, gender, race, and parental education (Table 1). Moreover, learning impairments likely reflect a confluence of cognitive, affective, and motivational processes, and their expression is shaped by environmental and task context. Future research could extend our approach to incorporate broader functional and affective dimensions to create a richer assessment environment that allows distinct probing of not only cognitive but also affective and motivational processes. This is important because what appears as a learning deficit in a cognitive task may instead reflect motivational disengagement or impulsivity in another task with social elements or reward-specific manipulations. Future studies could also explore associations between model-derived mechanisms and performance on independent cognitive tasks. To ensure interpretability, it will be important that such tasks are specifically designed to dissociate the underlying computational processes, rather than conflating multiple mechanisms under a single construct, as is often the case with classical WM tasks. Third, we did not observe reduced perseveration in MDD, which is in contrast to previous research (using other paradigms than the RL-WM task) albeit evidence remained mixed (47, 48, 49). It might be that the RL-WM task did not elicit the necessary strong affective reactions to reveal dysfunctional reward processing. Studies suggest that not only perseveration but also emotional reactivity and intensity are distinctly shaped by affective traits associated with mood disorders and anxiety disorders that frequently co-occur with MDD (50, 51, 52). Fourth, although individuals with SCZ, MDD, or BP often experience significant challenges in social interactions, these difficulties are underexplored in traditional neurocognitive testing (3,53, 54, 55, 56). Recent advances suggest that game theoretical paradigms from experimental economics, coupled with neurocomputational modeling, offer powerful tools to investigate latent mechanisms in social and strategic learning contexts (3,7,57,58).

Developing multidimensional assessments allows researchers to explore the mechanisms of learning and their interaction with cognitive flexibility, enhancing our understanding of behavioral adaptability across different cognitive and sociocognitive contexts (3,56). Behavioral adaptability is an essential clinical characteristic that often goes underexplored (1,56,59). This variability matters because mental states are dynamic and symptoms context sensitive; identifying its sources can inform personalized, adaptive interventions (60, 61).

## Supplementary Material

Supplemental Text, Figures S1–S9, and Tables S1–S18

Key Resources Table
