# Supplementary material for: Model-Based Electroencephalography Phenotyping Uncovers Distinct Neurocomputational Mechanisms Underlying Learning Impairments Across Psychopathologies
Source: Biol Psychiatry Glob Open Sci. 2025 Nov 29;6(2):100660. doi: 10.1016/j.bpsgos.2025.100660 (PMC12876727; doi:10.1016/j.bpsgos.2025.100660)
Supplement: Supplemental Text, Figures S1–S9, and Tables S1–S18 [file mmc1.pdf]

SUPPLEMENTARY INFORMATION

Model-Based Electroencephalography Phenotyping Uncovers Distinct Neurocomputational Mechanisms Underlying Learning Impairments Across Psychopathologies

Ging-Jehli *et al.*

1. Additional Study Information

Diagnostic Assessment. All participants completed a Structured Clinical Interview (SCID[1]) for DSM 5 with a trained researcher supervised by a doctoral-level clinician. Raters were trained with remote webinars in which rating scales and anchor points were discussed. Raters also completed and discussed a set of 6 training videos. Following this training, raters then worked to achieve consensus in their ratings with "gold standard" ratings that were supplied by experienced clinicians at the Maryland and St. Louis sites for at least 6 interviews. Consensus was defined as no more than 2 items with a difference of more than 1 rating point from the standard. To maintain inter-rater reliability over the course of the study, the St. Louis site recorded an interview to rate every 2-4 weeks, and all raters participated in remote meetings to resolve any discrepancies in ratings of this interview.

RLWM task. The RLWM task differentiates between RL and WM mechanisms by assessing the impact of distinct task manipulations on learning processes. Specifically, RL-related mechanisms are quantified by the increase in accuracy with the accumulation of reward history, defined as the number of previous correct responses for a given stimulus-response association. Hence, RL refers to the incremental learning process that evolves over time throughout the task. The task consisted of two phases: a learning phase and a subsequent reward retention test phase. For brevity, we report the main results of the reward retention phase in the Supplement, as they did not reveal clinically important findings.

RLWM modeling. Initial work with this model assumed that the two modules were independent, in part because individual variations in RL and WM parameters were associated with genetic components affecting striatal plasticity and prefrontal function, respectively.[2] More recent studies have shown, however, WM and RL systems also interact *cooperatively*.[3–6] In particular,

these studies have shown that the RPEs needed for learning by the RL system are influenced by expectations that are held in WM. Consider, for example, a trial in which participants respond correctly and get positive feedback. When stimulus-outcome associations can be held in WM (during low WM load), this correct outcome will match WM-based expectations, reducing the RPE experienced by the RL module (Fig. 1C). Conversely, under high WM demand, outcomes are not reliably expected in WM, and accordingly, larger RPEs cause increased Q-value updating in RL, reducing the burden on the capacity-limited WM module. This cooperative tradeoff is supported by fMRI, EEG and behavioral studies.[3–6] Importantly, these predictions do not imply that *either* RL *or* WM processes are recruited on a given trial but rather they interact dynamically within and across trials. This phenomenon provides an opportunity to study how these interactions might vary in the clinical conditions studied here.

EEG recording and processing. Although participants were recruited from different sites, we used a shared infrastructure and standardized testing protocols as well as analyses pipelines. Specifically, all behavioral and EEG data were collected using the same task implementation, hardware setup, EEG system and preprocessing pipeline. Testing procedures were harmonized across sites, including experimenter training and instructions to participants, and data quality was formally assessed on a regular basis to ensure similarity across sites.[7]

The continuous EEG was down-sampled to 125Hz, then was reduced to a selected window of -100 to +700ms twice. Once it was epoched and baseline corrected from –100 to 0ms before the onset of the stimulus and second epoched and baseline corrected from –100 to 0ms before the onset of the feedback. The epoched data was subjected to an artifact detection algorithm (100µV voltage threshold with a moving window width of 200ms and a 100ms window step) followed by

manual verification. Trials containing large artifacts were flagged and removed later at the univariate EEG analysis stage.

Corrected ERPs. We computed the predicted voltage using the multiple-regression model described above while setting a single regressor to 0 (set size, delay, expected Q value, or reaction time); we subtracted this predicted voltage from the true voltage (for every electrode and time point within each trial), leaving only the fixed effect, the variance explained by that regressor, and the residual noise of the regression model. ERPs were computed as the average corrected voltage from all trials that belong to the same level of condition. Note that the array of expected Q values was divided to 4 quartiles and trials within each quartile were averaged for plotting ERPs.

Trial-by-trial similarity index of WM and RL. A multiple regression analysis was conducted for each participant, in which the EEG amplitude at each electrode site and time point was predicted by the conditions of interest (set size, delay, model-derived RL expected value, and their interactions). The delay predictor (the number of trials since the stimulus was presented and a correct response was made) used in the regression analyses was inverse transformed (-1/delay) to avoid the disproportion effect of very large but rare delays. We used the previously identified analysis method[4,5] to identify spatiotemporal clusters (masks) of the three main predictors in the GLM (set-size, delay, and model-derived RL expected value). Specifically, we tested the significance of each time point at each electrode across participants against 0 using only trials with correct responses.

Importantly, as in previous research, we restricted our analysis to correct responses because we are interested in the underlying process of incremental learning. Focusing on correct responses then shows the dynamics of successful learning. This also avoids confounding effects due to positive or negative RPEs. In that sense, the RPE effects represent surprise and sensitivity to

correct responses. This is because the actual percept a participant sees on the screen is the same on each trial. Hence, the only difference across trials is their learned expectations about reward. This is also why we expect it to be larger in high set size and systematically go down with experience. This approach ensures that all variance in the signal has to do with expectations, since that is the only thing that differs**.**

For each marker we control what is presented to the subject. For instance, during choice, participants always look at one stimulus and prepare a response, but the neural WM markers reflect how many other stimuli they are learning about and how long it has been since they saw the current one; and the neural RL-Q marker reflects the reward expectation for that option based on their reward history and estimated learning rate. Neural WM markers reflect the degree to which a given stimulus elicits brain activity related to the need to manage multiple concurrent stimuli in WM.

Cluster statistics. We used cluster-mass correction by permutation testing with custom-written Matlab scripts for statistical inference. The spatiotemporal cluster mask is defined by the significant data points connected by temporal adjacency. The threshold for a t-test significance level for each time point is defined as $P < (0.001)$. This was repeated 1000 times, generating a distribution of maximum cluster-mass statistics under the null hypothesis. Only clusters with a greater t-value sum than the maximum cluster mass obtained with 97.5% chance permutations were considered significant. Before using the mask to compute the similarity index in each trial we refined the mask and maintained only the highest effect size data points (10th percentile) within each mask. We then assessed each trial's neural similarity to the spatiotemporal mask of the condition by computing the dot product between the spatiotemporal voltage activity map of the individual trial and the spatiotemporal t-value map of the mask. This computation produced a trial-

level similarity measure intended to assess the trial-wise experienced WM load and delay effects, as well as trial-wise RL contributions.

Model fitting procedure. We evaluated model fit at the individual level by computing the negative log-likelihood (NLL) of the fitted model for each subject, and derived the average likelihood per trial using the referenced formula. Subjects performing at or below chance (e.g., likelihood ~0.33) were flagged as potential non-compliant or poorly fit. None of the participants fell into this category (the highest value for one subject was found to be equal to 0.3).

Reward retention test phase. Stimuli that had been rewarded more often during learning were selected more often as having higher reward value in the test phase [ΔQ: $\beta = 1.11$, $SE = 0.09$, $p < 0.001$]. Moreover, performance in this test phase was enhanced when stimulus values were learned under higher than lower set size [mean(ss)-by-ΔQ: $\beta = 0.09$, $SE = 0.03$, $p = 0.001$] which is consistent with past findings.[5,6] This counterintuitive result is consistent with the EEG findings above and the WMRL interaction model, whereby larger RPEs during high WM load induce more robust RL computations that are expressed during the retention phase.

Clinical differences in reward retention testing. Data showed consistent performance at the test phase across clinical groups (p>.1) Note that we also controlled for response perseveration; no significant tendency was observed for repeating the same response used in the previous trial ($p > 0.20$).

Sensitivity analyses of group differences in RLWM parameters after outlier removal. To assess the robustness of the reported group differences in RLWM parameters (see Fig. S7), we conducted sensitivity analyses in which outliers were removed prior to re-analysis. Outliers were identified separately within each diagnostic group using the 1.5 x interquartile range (IQR) rule. Specifically, for each parameter, values falling below the first quartile minus 1.5×IQR or above

the third quartile plus 1.5 x IQR were flagged as outliers. This non-parametric approach was chosen for its robustness to skewed distributions and group-specific variability. Participants with outlier values on any parameter were excluded from this secondary analysis. This procedure resulted in the exclusion of 56 participants (CTRL = 19, BP = 15, MDD = 5, SCZ = 17). The resulting parameter distributions are shown in Fig. S8. Hence, removal of outliers did not alter the pattern of group differences reported in Fig. S7 or in the final section of the Results in the main manuscript. These findings suggest that the reported effects are not driven by extreme values and are robust to outlier exclusion. We also repeated the between-subject correlation analyses reported in the last subsection of the Results, this time excluding participants identified as outliers based on model parameters. The findings remained robust: First, within the BP group, participants with greater working memory decay (higher φ) continued to show reduced neural reinforcement learning markers ($r = -0.48$, $p = 0.006$). Second, within the MDD group, participants who relied more on working memory than reinforcement learning processes (higher ρ) still exhibited reduced neural set size markers ($r = -0.35$, $p = 0.021$).

Model-based EEG analyses. Although we did not examine spectral dynamics directly, the model-based EEG approach used here isolates neural markers of latent RL and WM computations without relying on predefined frequency bands or specific event-related components but rather focuses on extracting the latent mechanisms are simulated by the computational model. Prior work has shown that such voltage-based regressors yield robust signals of underlying computational processes, though future work may integrate both time- and frequency-domain analyses to further enrich mechanistic interpretations.

2. Supplementary Figures

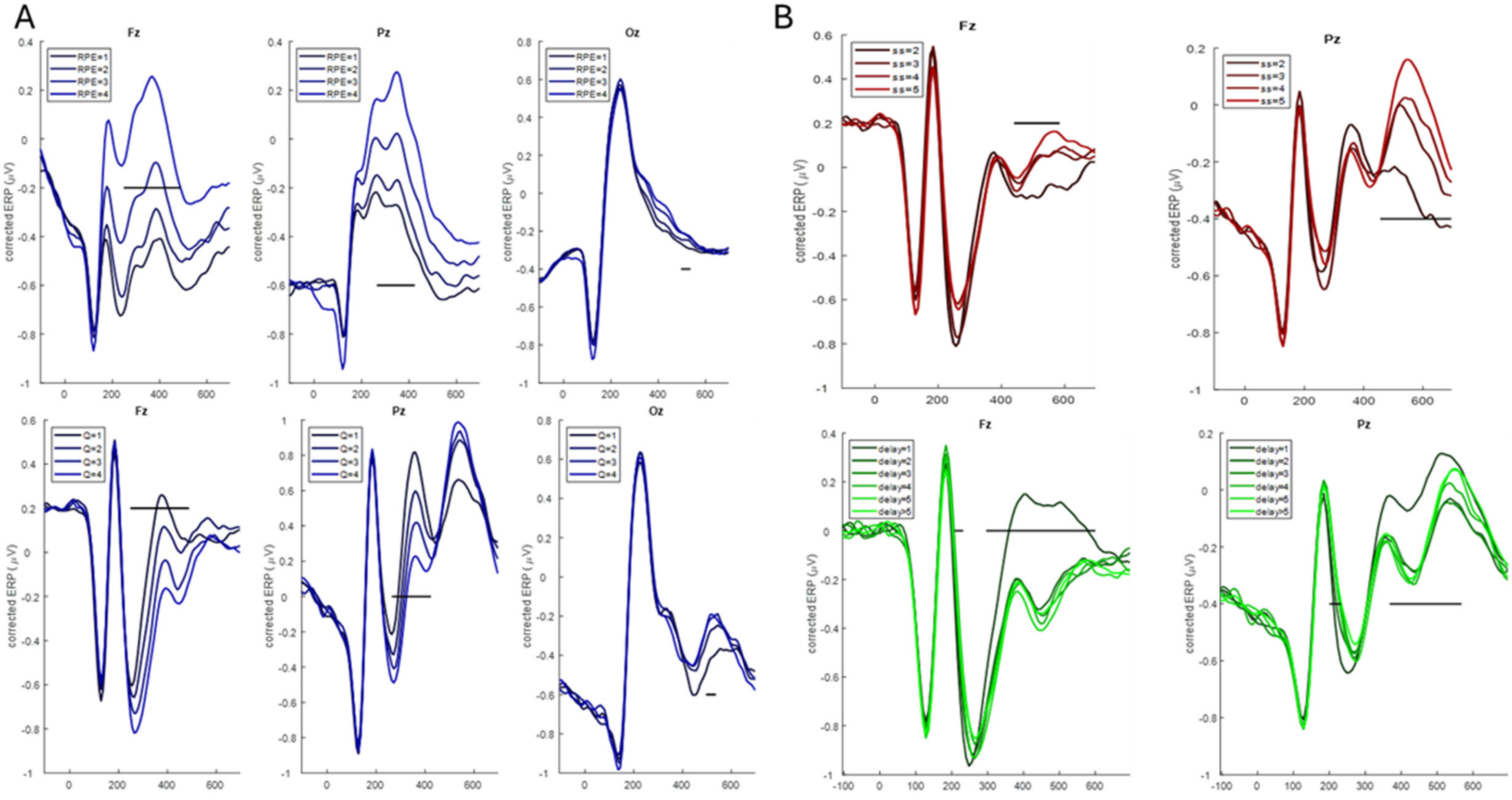

Fig. S1. Event-related potentials (ERPs) by markers of reinforcement learning (RL) and working memory (WM).

(A) ERP plots show the effect of the extracted RL markers, reward prediction errors (RPEs) and Q-values, derived from computational modeling and GLM (detailed in the Methods). Shown are the time course for four quartile values. Horizontal black lines reflect significant time points after permutation correction. **(B)** ERP plots show the effect of working memory markers (set size and delay) on the voltage of significant electrodes (Fz and Pz both at central lines). Horizontal black lines reflect significant time points after permutation correction.

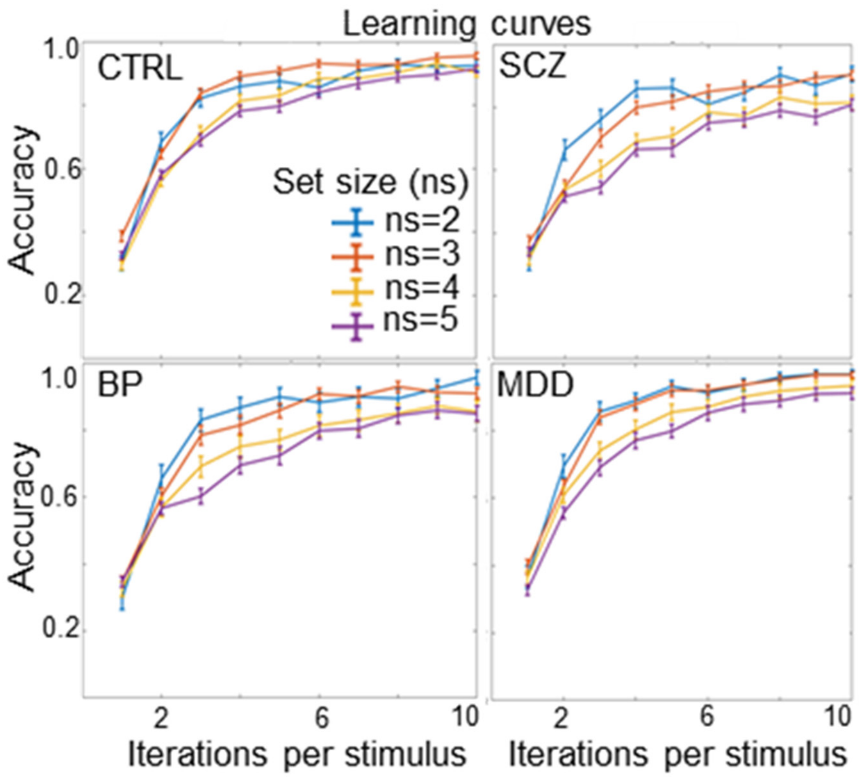

Fig. S2. Learning curves by clinical group.

Accuracy curves (averaged across trials, participants, and groups) by number of stimulus iterations show continuous learning over time that varies with set size in all clinical groups. Vertical bars represent within-subject SEs. CTRL refers to participants without any mental health diagnoses; SCZ refers to participants diagnosed with schizophrenia; BP refers to participants diagnosed with bipolar disorder; MDD refers to participants diagnosed with major depressive disorders.

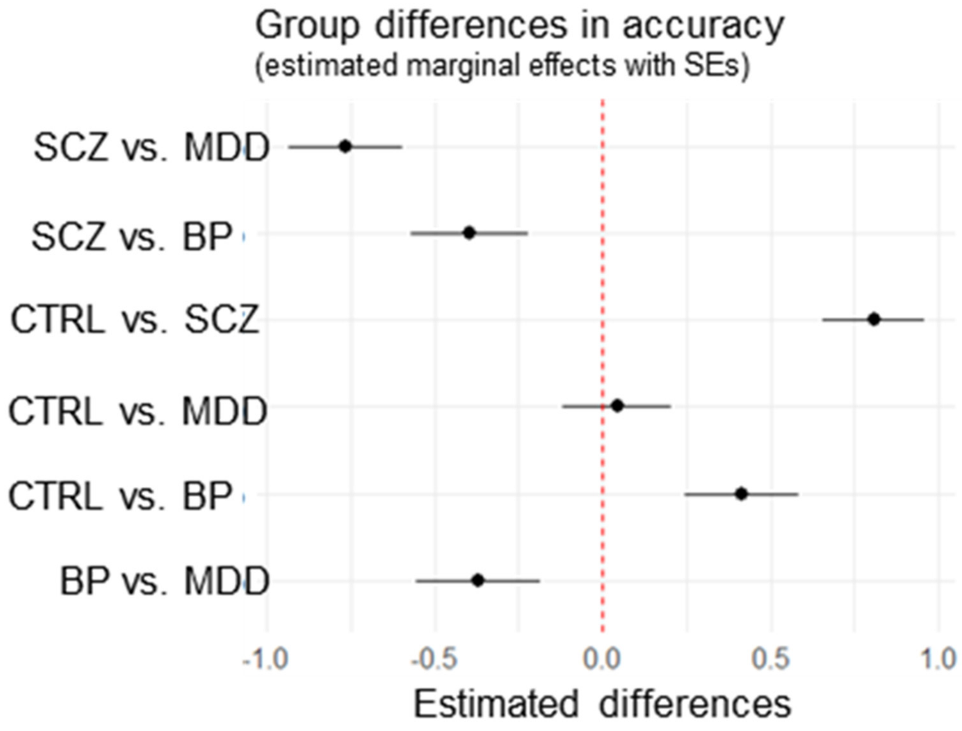

Fig. S3. Group-wise comparison in overall accuracy (across all trials).

Results from regression-based models with accuracy (across all trials) as dependent variable and group as independent variable. Points refer to means and horizontal lines refer to estimated standard errors. The CTRL and MDD group had an overall higher accuracy than the SCZ and BP groups. The CTRL and MDD group did not differ in overall accuracy. The BP group had a higher accuracy than the SCZ group. CTRL refers to participants without any mental health diagnoses; SCZ refers to participants diagnosed with schizophrenia; BP refers to participants diagnosed with bipolar disorder; MDD refers to participants diagnosed with major depressive disorders.

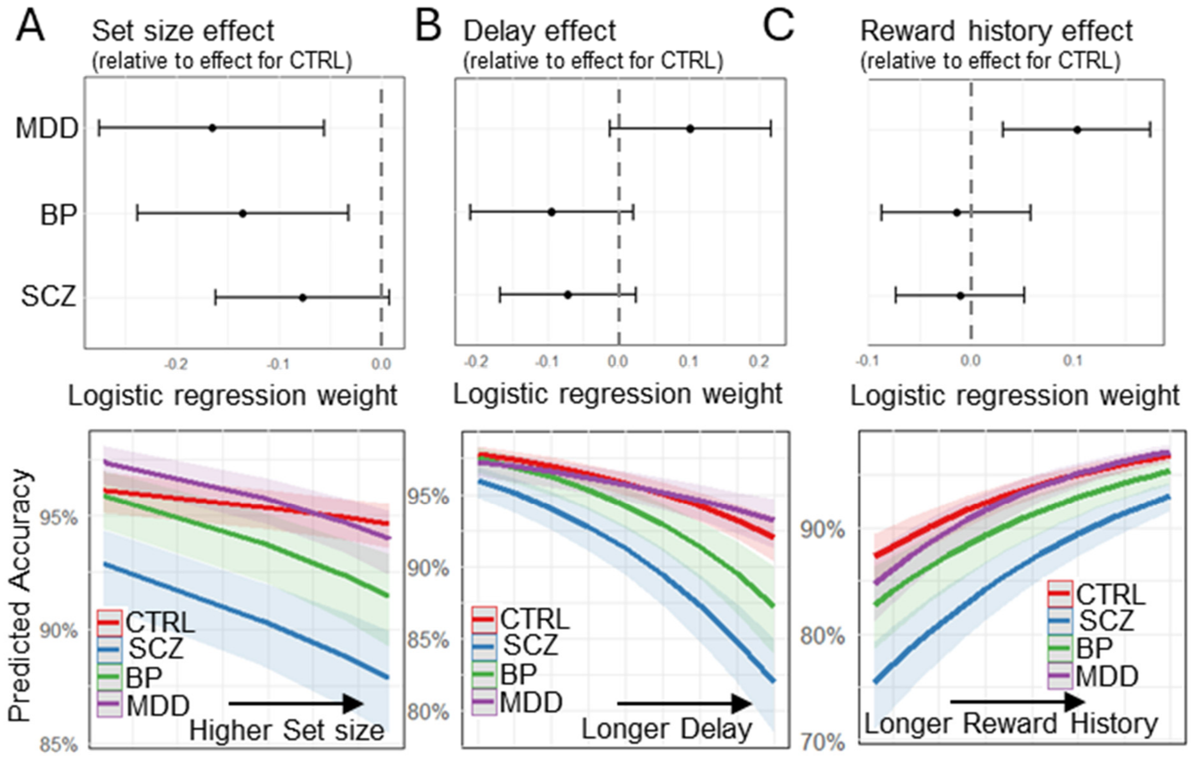

Fig. S4. Group differences in set size, delay, and reward history effects.

Results from logistic regression with accuracy as dependent variable and predictors including delay, set size, reward history, group and their interactions. Dots represent point estimates and lines represent 95%-CI. This analysis showed larger set size effects for MDD and BP compared to CTRL **(A)** as well as smaller reward history effects for MDD compared to CTRL **(C)**. We did not find significant group differences in delay effects **(B)**. See Suppl. Table S10 for regression output. CTRL refers to participants without any mental health diagnoses; SCZ refers to participants diagnosed with schizophrenia; BP refers to participants diagnosed with bipolar disorder; MDD refers to participants diagnosed with major depressive disorders.

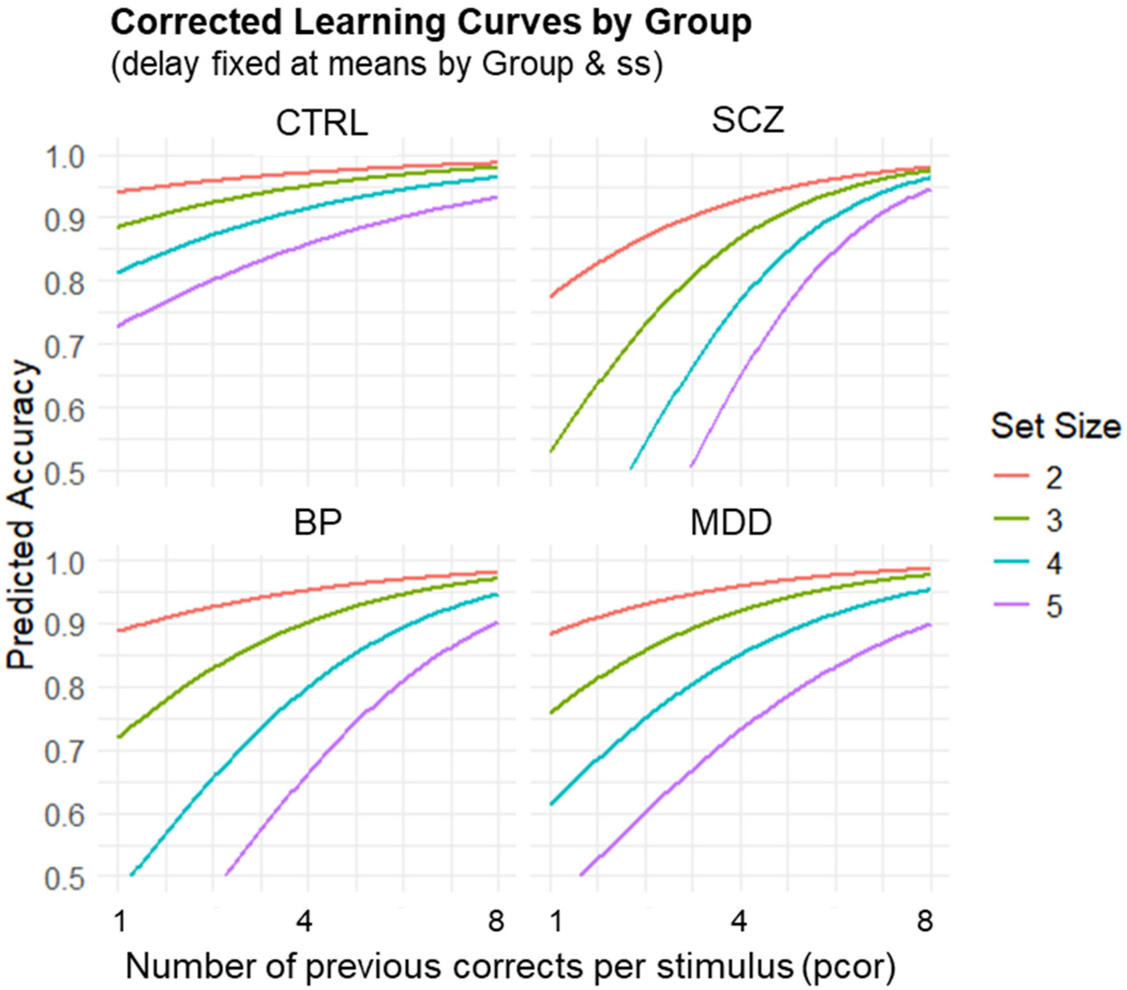

Fig. S5. Delay-corrected learning curve by group.

Graphical illustration of the pronounced set size effect specific to MDD, BP, and SCZ with corrected learning curves by fixing delay at mean level within each set size and group. We also estimated a regression with accuracy as dependent variable and predictors including group, set size, delay, reward history, and their interactions. Compared to CTRL, BP and MDD had more trouble maintaining accuracy as set size increased across blocks. CTRL refers to participants without any mental health diagnoses; SCZ refers to participants diagnosed with schizophrenia; BP refers to participants diagnosed with bipolar disorder; MDD refers to participants diagnosed with major depressive disorders.

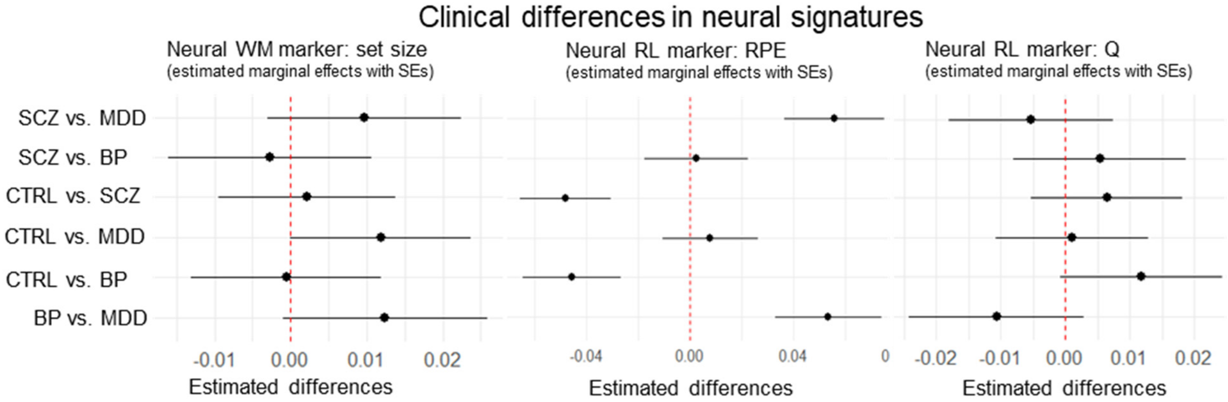

Fig. S6. Group differences in neural markers of working memory (WM) and reinforcement learning (RL).

Results from regression-based models with neural WM markers (set size) and reinforcement learning markers (RPEs, Q-values) as dependent variable and group as independent variable. We do not show neural markers of delay effects since we did not find significant group differences (see Supplementary Fig. S4B). Points refer to means and horizontal lines refer to estimated standard errors. **(A)** The CTRL group had overall higher neural set size markers than the MDD group. **(B)** The SCZ and BP groups had higher neural RPE markers than the CTRL and MDD groups. **(C)** The CTRL group had slightly higher neural Q markers than the CTRL group. CTRL refers to participants without any mental health diagnoses; SCZ refers to participants diagnosed with schizophrenia; BP refers to participants diagnosed with bipolar disorder; MDD refers to participants diagnosed with major depressive disorders.

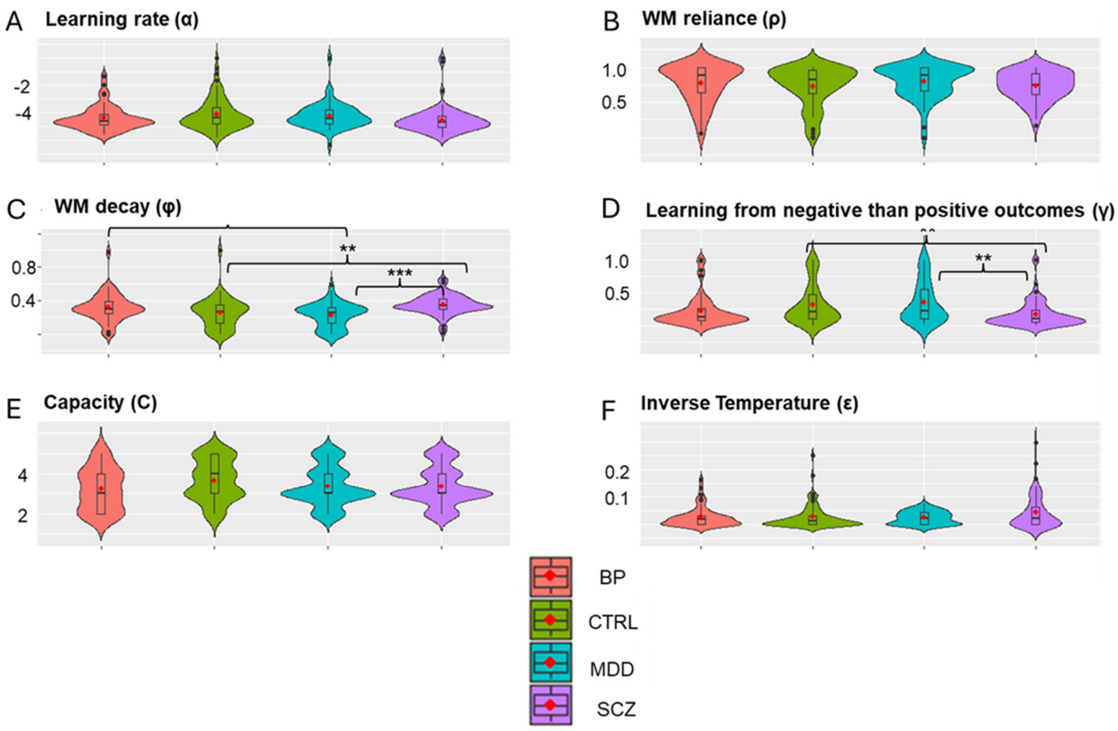

Fig. S7. Group differences in computational RLWM model parameters.

Shown are distribution of RLWM parameters with the red dots representing group means and the vertical boxplots representing the median (thick horizontal lines) and interquartile ranges. CTRL refers to participants without any mental health diagnoses; SCZ refers to participants diagnosed with schizophrenia; BP refers to participants diagnosed with bipolar disorder; MDD refers to participants diagnosed with major depressive disorders. Note that the parameter β is fixed at a value of 100 to prevent significant trade-offs with other parameters during model fitting. For details, see Method section in the main manuscript. **(A-B)** Shown are the distribution of RLWM model parameter by group for **(A)** learning rate (α) and **(B)** WM reliance (ρ) for which we did not find any significant group differences. **(C)** Distribution of RLWM model parameter φ (WM decay) by group. The one-way ANOVA suggested a statistically significant difference in model parameter between the groups, $F(3, 251) = 8.020$, $p < 0.001$. To identify which groups differed significantly, a Tukey's Honest Significant Difference (HSD) post-hoc test was performed. Significant results of the Tukey HSD test were: 1. Mean(MDD) minus Mean(BP) = -0.087, *95%-CI* = [-0.168, -0.006], *p-adjusted* = 0.030; 2. Mean(SCZ) minus Mean(CTRL) = 0.096, *95%-CI* = [0.031, 0.163], *p-adjusted* = 0.001; 3. Mean(SCZ) minus Mean(MDD) = 0.123, *95%-CI* = [0.123, 0.049], *p-adjusted* < 0.001. **(D)** Distribution of RLWM model parameter (γ) by group. This parameter indexes learning more from negative than positive outcomes and is estimated by applying the RLWM computational model to accuracy as task performance measure. The one-way ANOVA suggested a statistically significant difference in model parameter between the groups, $F(3, 251) = 6.058$, $p < 0.001$. To identify which groups differed significantly, a Tukey's Honest Significant Difference (HSD) post-hoc test was performed. Significant results of the Tukey HSD test were: 1. Mean(SCZ) minus Mean(CTRL) = -0.144, *95%-CI* = [-0.256, -0.031], *p-adjusted* = 0.006; 2. Mean(SCZ) minus Mean(MDD) = -0.178, *95%-CI* = [-0.305, -0.052], *p-adjusted* = 0.002. **(E-F)** Shown are the distribution of RLWM model parameter by group for **(E)** capacity (C) and **(F)** inverse temperature (ε) for which we did not find any significant group differences.

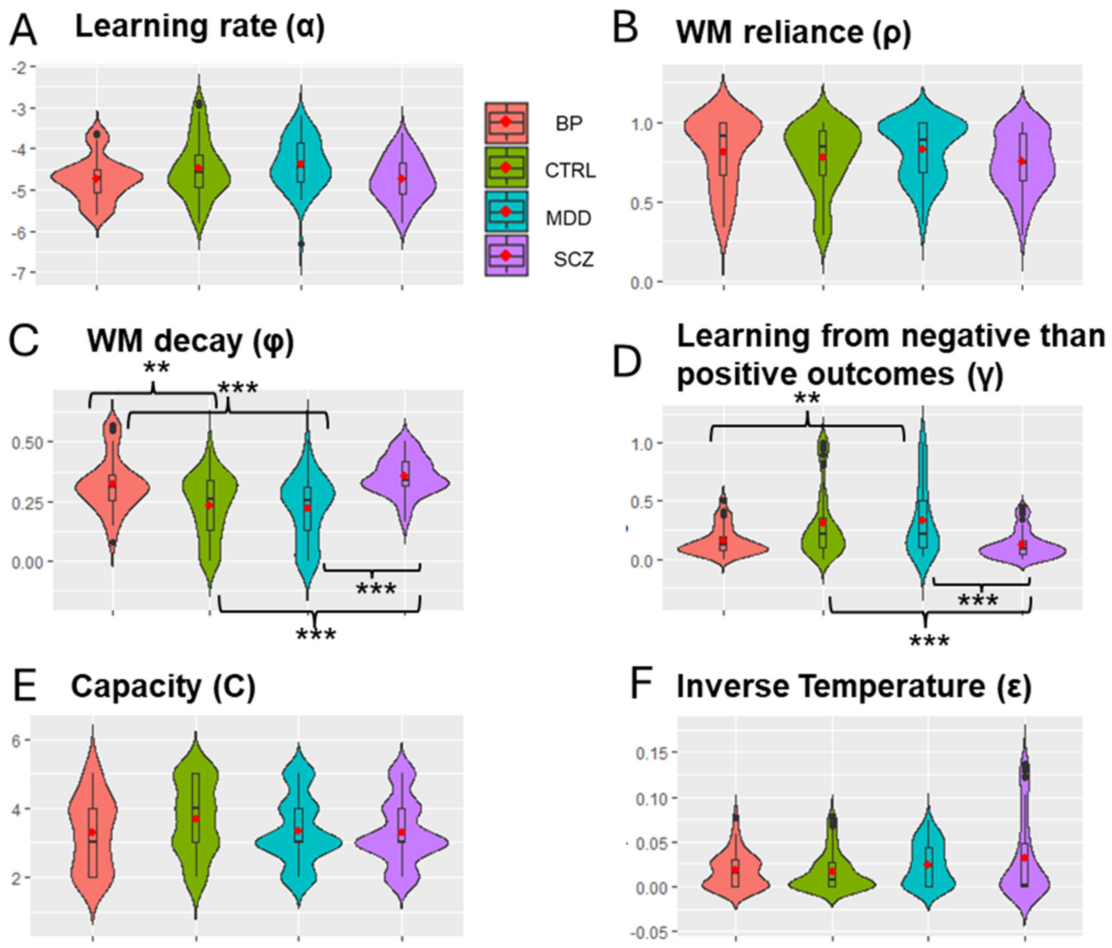

Fig. S8. Group differences in RLWM model parameters after outlier removal.

Shown are distribution of RLWM parameters with the red dots representing group means and the vertical boxplots representing the median (thick horizontal lines) and interquartile ranges. CTRL refers to participants without any mental health diagnoses; SCZ refers to participants diagnosed with schizophrenia; BP refers to participants diagnosed with bipolar disorder; MDD refers to participants diagnosed with major depressive disorders. Note that the parameter β is fixed at a value of 100 to prevent significant trade-offs with other parameters during model fitting. For details, see Method section in the main manuscript. **(A-B)** Shown are the distribution of RLWM model parameter by group for **(A)** learning rate (α) and **(B)** WM reliance (ρ) for which we did not find any significant group differences. **(C)** Distribution of RLWM model parameter φ (WM decay) by group. The one-way ANOVA suggested a statistically significant difference in model parameter between the groups, $F(3, 195) = 16.22$, $p < 0.001$. To identify which groups differed significantly, a Tukey's Honest Significant Difference (HSD) post-hoc test was performed. Significant results of the Tukey HSD test were: 1. Mean(CTRL) minus Mean(BP) = -0.089, *95%-CI* = [-0.154, -0.023], *p-adjusted* = 0.003; 2. Mean(MDD) minus Mean(BP) = -0.101, *95%-CI* = [-0.171, -0.032], *p-adjusted* = 0.001; 3. Mean(SCZ) minus Mean(CTRL) = 0.124, *95%-CI* = [0.067, 0.181], *p-adjusted* < 0.001; 4. Mean(SCZ) minus Mean(MDD) = 0.137, *95%-CI* = [0.075, 0.198], *p-adjusted* < 0.001. **(D)** Distribution of RLWM model parameter (γ) by group. This parameter indexes learning more from negative than positive outcomes and is estimated by applying the RLWM computational model to accuracy as task performance measure. The one-way ANOVA suggested a statistically significant difference in model parameter between

the groups, $F(3, 195) = 9.515$, $p < 0.001$. To identify which groups differed significantly, a Tukey's Honest Significant Difference (HSD) post-hoc test was performed. Significant results of the Tukey HSD test were: 1. Mean(MDD) minus Mean(BP) = 0.181, *95%-CI* = [0.037, 0.324], *p-adjusted* = 0.007; 2. Mean(SCZ) minus Mean(CTRL) = -0.187, *95%-CI* = [-0.304, -0.069], *p-adjusted* < 0.001; 3. Mean(SCZ) minus Mean(MDD) = -0.212, *95%-CI* = [-0.339, -0.086], *p-adjusted* < 0.001. **(E-F)** Shown are the distribution of RLWM model parameter by group for **(E)** capacity (C) and **(F)** inverse temperature (ε) for which we did not find any significant group differences.

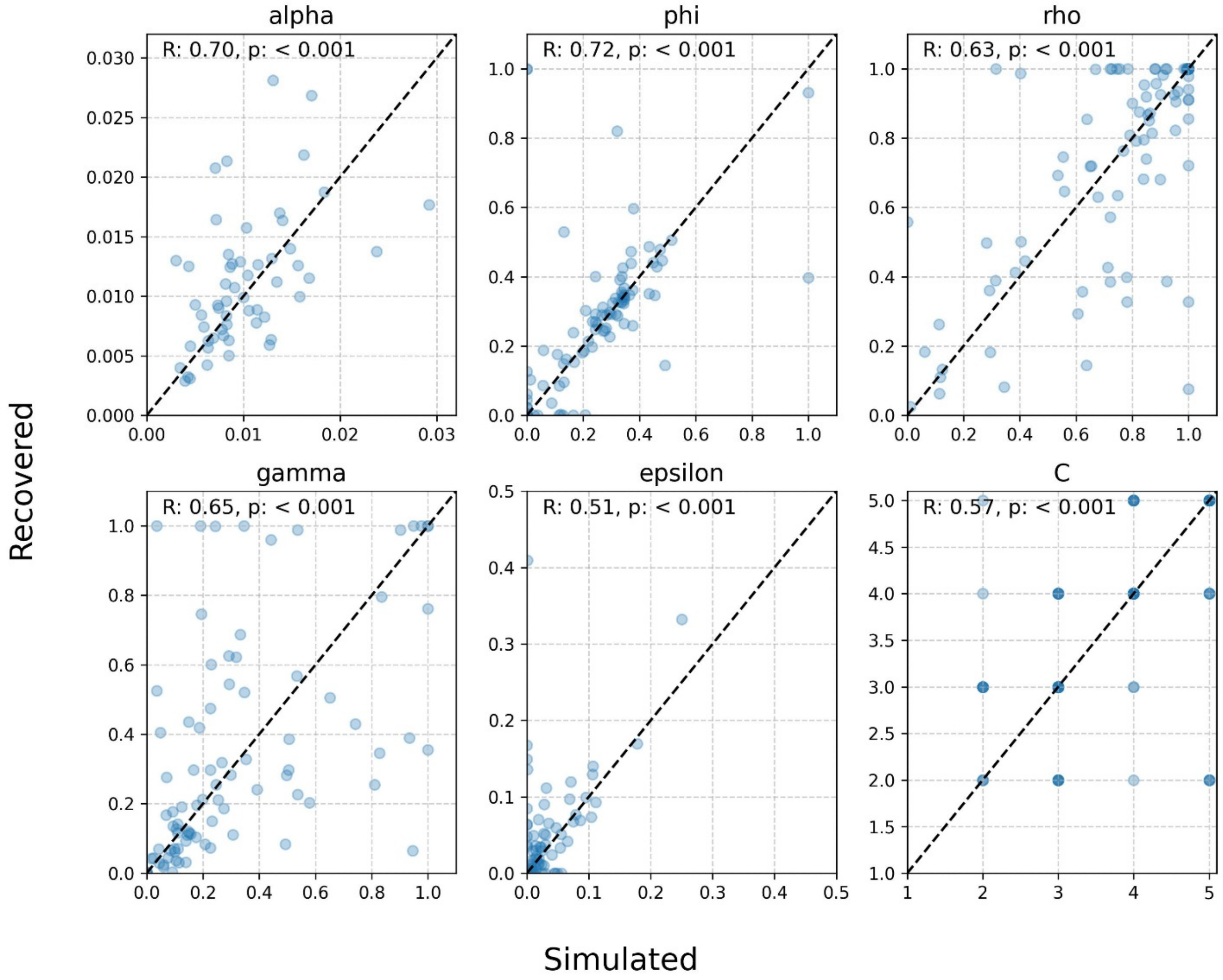

Fig. S9. Parameter recovery based on simulated data.

Results from simulations comparing input parameters (x-axis) and recovered parameters (y-axis). Overall, these plots show good parameter recovery (R refers to correlation coefficients).

3. Supplementary Tables

Table S1. Regression output of model with accuracy as dependent variable.

**Dependent variable: Accuracy**			
Predictors	*Odds Ratios*	*CI*	*p*
(Intercept)	16.69	14.52 – 19.17	**<0.001**
Set Size	0.75	0.70 – 0.80	**<0.001**
Delay	0.77	0.72 – 0.82	**<0.001**
Pcor	1.64	1.58 – 1.69	**<0.001**
Set Size × Delay	0.67	0.64 – 0.70	**<0.001**
Set Size × Pcor	1	0.95 – 1.04	0.843
Delay × Pcor	1.25	1.20 – 1.31	**<0.001**
**Random Effects**			
σ^2	3.29		
$\tau_{00\ sbj}$	1.14		
$\tau_{11\ sbj.SetSize}$	0.12		
$\tau_{11\ sbj.Delay}$	0.09		
$\tau_{11\ sbj.Pcor}$	0.02		
$\tau_{11\ sbj.SetSize:Delay}$	0.02		
$\tau_{11\ sbj.SetSize:Pcor}$	0.02		
$\tau_{11\ sbj.Delay:Pcor}$	0.01		
ρ_{01}	-0.1		
	0.58		
	0.35		
	-0.38		
	0.08		
	0.56		
N_{sbj}	255		
Observations	68475		
Marginal R^2	0.154		

Output from multi-linear mixed regression model fitted across all participants, complementing regression coefficient in Figure 2B of main manuscript. Regression model included accuracy as binary dependent variable, corrects (1) and errors (0), delay, and previous correct responses indexing reward history (pcor) as well as their two way interactions as independent variables. Shown are means and 95% confidence intervals of fixed effects as well as estimated random effects.

Table S2. Regression output of model with EEG-Q as dependent variable.

**Dependent variable: EEG-Q**			
Predictors	*Estimates*	*CI*	*p*
(Intercept)	0.05	0.05 – 0.06	**<0.001**
Set Size	0	-0.01 – 0.01	0.776
Delay	0.05	0.04 – 0.05	**<0.001**
Pcor	0.11	0.10 – 0.12	**<0.001**
Set Size × Delay	-0.01	-0.01 – 0.00	0.191
Set Size × Pcor	0	-0.01 – 0.01	0.947
Delay × Pcor	0.01	-0.00 – 0.02	0.06
**Random Effects**			
σ^2	0.98		
$\tau_{00\ sbj}$	0		
N_{sbj}	222		
Observations	51794		
Marginal R^2	0.014		

Output from multi-linear mixed regression model fitted across all participants, complementing regression coefficient in Figure 2D of main manuscript. Mixed-effect regressions included neural RL markers as dependent variable and predictors: set size, delay, reward history (pcor), and their interactions. Shown are means and 95% confidence intervals of fixed effects as well as estimated random effects.

Table S3. Regression output of model with EEG-RPE as dependent variable.

**Dependent variable: EEG-RPE**			
Predictors	*Estimates*	*CI*	*p*
(Intercept)	-0.16	-0.17 – -0.15	**<0.001**
Set Size	0.06	0.05 – 0.07	**<0.001**
Delay	0.03	0.02 – 0.04	**<0.001**
Pcor	-0.17	-0.18 – -0.16	**<0.001**
Set Size × Delay	0.04	0.03 – 0.04	**<0.001**
Set Size × Pcor	-0.02	-0.03 – -0.01	**<0.001**
Delay × Pcor	-0.05	-0.06 – -0.04	**<0.001**
**Random Effects**			
σ^2	0.93		
$\tau_{00\ sbj}$	0.01		
N_{sbj}	222		
Observations	51794		
Marginal R^2	0.039		

Output from multi-linear mixed regression model fitted across all participants, complementing regression coefficient in Figure 2F of main manuscript. Mixed-effect regressions included neural reward prediction error (RPE) markers as dependent variable and predictors: set size, delay, reward history (pcor), and their interactions. Shown are means and 95% confidence intervals of fixed effects as well as estimated random effects.

Table S4. Regression output of model with EEG-Q as dependent variable.

**Dependent variable: EEG-Q**			
Predictors	*Estimates*	*CI*	*p*
(Intercept)	0	-0.01 – 0.01	0.992
Pcor	0.12	0.11 – 0.13	**<0.001**
Neural set size marker	-0.33	-0.35 – -0.32	**<0.001**
Set Size	0.05	0.04 – 0.07	**<0.001**
Pcor × Neural set size marker	0.01	0.00 – 0.02	**0.02**
Pcor × Set Size	0	-0.00 – 0.01	0.382
Neural set size marker × Set Size	0	-0.01 – 0.01	0.515
Pcor × Neural set size marker × Set Size	-0.01	-0.01 – 0.00	0.198
**Random Effects**			
σ^2	0.86		
$\tau_{00\ sbj}$	0		
$\tau_{11\ sbj.Pcor}$	0		
$\tau_{11\ sbj.NeuralSetSizeMarker}$	0.01		
$\tau_{11\ sbj.SetSize}$	0.01		
$\tau_{11\ sbj.Pcor\_by\_NeuralSetSizeMarker}$	0		
$\tau_{11\ sbj.Pcor\_by\_SetSize}$	0		
$\tau_{11\ sbj.SetSize\_by\_NeuralSetSizeMarker}$	0		
$\tau_{11\ sbj.Pcor\_by\_SetSize\_by\_NeuralSetSizeMarker}$	0		
ρ_{01}	0.2		
	0.99		
	0.19		
	0.22		
	0.05		
	-0.13		
	-0.02		
N_{sbj}	222		
Observations	51794		
Marginal R^2	0.125		

Output from multi-linear mixed regression model fitted across all participants to separate WM and RL contributions, we estimated a linear mixed-effects regression with neural RL markers as dependent variable and predictors including behavioral and neural WM components (set size, neural set size markers), RL components (reward history), and their interactions. Shown are means and 95% confidence intervals of fixed effects as well as estimated random effects.

Table S5. Regression output of model with EEG-SetSize as dependent variable.

**Dependent variable: EEG-SetSize**			
Predictors	*Estimates*	*CI*	*p*
(Intercept)	0	-0.01 – 0.01	0.936
Set Size	0.12	0.10 – 0.13	**<0.001**
Pcor	0.02	0.01 – 0.03	**<0.001**
Set Size × Pcor	0.01	0.00 – 0.02	**0.015**
**Random Effects**			
σ^2	0.97		
$\tau_{00\ \mathrm{sbj}}$	0		
$\tau_{11\ \mathrm{sbj.SetSize}}$	0		
$\tau_{11\ \mathrm{sbj.Pcor}}$	0		
$\tau_{11\ \mathrm{sbj.SetSize\_by\_Pcor}}$	0		
ρ_{01}	0		
	0		
	0		
N_{sbj}	222		
Observations	51794		
Marginal R^2	0.014		

Output from multi-linear mixed regression model fitted across all participants. Model included neural set size marker as dependent variable and predictors: set size, reward history (pcor), and their interactions. Shown are means and 95% confidence intervals of fixed effects as well as estimated random effects.

Table S6. Regression output of model with Accuracy as dependent variable.

**Dependent variable: Accuracy**			
Predictors	*Odds Ratios*	*CI*	*p*
(Intercept)	12.22	10.65 – 14.01	**<0.001**
Set size	0.79	0.76 – 0.81	**<0.001**
Neural set size marker	1.09	1.06 – 1.12	**<0.001**
Pcor	1.71	1.67 – 1.75	**<0.001**
Set size × neural set size marker	1.04	1.01 – 1.08	**0.009**
Set size × Pcor	1.07	1.04 – 1.10	**<0.001**
Neural set size marker × Pcor	1.03	1.00 – 1.05	**0.035**
Set size × neural set size marker × Pcor	1.04	1.01 – 1.07	**0.003**
**Random Effects**			
σ^2	3.29		
$\tau_{00\ sbj}$	1.01		
N_{sbj}	222		
Observations	58381		
Marginal R^2	0.081		

Output from multi-linear mixed regression model fitted across all participants. Model included accuracy as dependent variable and predictors: set size, neural set size marker, reward history (pcor), and their interactions. Shown are means and 95% confidence intervals of fixed effects as well as estimated random effects.

Table S7. Regression output of model with EEG-RPE as dependent variable.

**Dependent variable: EEG-RPE**			
Predictors	*Estimates*	*CI*	*p*
(Intercept)	0	-0.01 – 0.01	1
Neural Q marker	-0.03	-0.04 – -0.02	**<0.001**
RPE	-0.07	-0.09 – -0.06	**<0.001**
**Random Effects**			
σ^2	0.98		
$\tau_{00\ sbj}$	0		
$\tau_{11\ sbj.NeuralQmarker}$	0		
$\tau_{11\ sbj.RPE}$	0.01		
ρ_{01}	0		
	0		
N_{sbj}	222		
Observations	58381		
Marginal R^2	0.006		

Output from multi-linear mixed regression model fitted across all participants. Model included neural reward prediction error (RPE) marker as dependent variable and predictors: RPE (estimated from the RLWM computational model as described in the main manuscript) and neural Q marker. Shown are means and 95% confidence intervals of fixed effects as well as estimated random effects.

Table S8. Regression output of model with EEG-RPE as dependent variable.

	Dependent variable		
Predictors	*Estimates*	*CI*	*p*
(Intercept)	0	-0.01 – 0.01	0.888
Set size	0.07	0.06 – 0.07	**<0.001**
Neural set size marker	-0.01	-0.02 – -0.00	**0.036**
Set size × neural set size marker	-0.01	-0.01 – 0.00	0.233
**Random Effects**			
σ^2	0.99		
$\tau_{00\ sbj}$	0		
N_{sbj}	222		
Observations	51794		
Marginal R^2	0.004		

Output from multi-linear mixed regression model fitted across all participants. Model included neural reward prediction error (RPE) marker as dependent variable and predictors: set size, neural set size marker, and their interactions. Shown are means and 95% confidence intervals of fixed effects as well as estimated random effects.

Table S9. Regression output of clinical model with accuracy as dependent variable.

**Dependent variable: Accuracy**			
Predictors	*Odds Ratios*	*CI*	*p*
(Intercept)	16.24	12.90 – 20.45	**<0.001**
Set size	0.92	0.87 – 0.98	**0.01**
Neural set size marker	1.08	1.02 – 1.15	**0.008**
Delay	0.75	0.70 – 0.80	**<0.001**
Pcor	1.59	1.52 – 1.67	**<0.001**
Group [SCZ]	0.47	0.34 – 0.66	**<0.001**
Group [BP]	0.73	0.50 – 1.06	0.097
Group [MDD]	1.17	0.81 – 1.68	0.402
Set size × neural set size marker	1.08	1.02 – 1.15	**0.014**
Neural set size marker × Delay	0.94	0.88 – 1.00	**0.043**
Neural set size marker × Pcor	1.01	0.96 – 1.06	0.665
Delay × Pcor	1.2	1.14 – 1.28	**<0.001**
Set size × Group [SCZ]	0.92	0.85 – 1.00	**0.05**
Set size × Group [BP]	0.86	0.77 – 0.95	**0.002**
Set size × Group [MDD]	0.95	0.85 – 1.05	0.285
Neural set size marker × Group [SCZ]	0.99	0.91 – 1.07	0.766
Neural set size marker × Group [BP]	1	0.91 – 1.10	0.972
Neural set size marker × Group [MDD]	0.96	0.86 – 1.06	0.388
Delay × Group [SCZ]	0.9	0.82 – 0.99	**0.029**
Delay × Group [BP]	0.85	0.76 – 0.95	**0.004**
Delay × Group [MDD]	1.14	1.02 – 1.27	**0.018**
Pcor × Group [SCZ]	0.97	0.91 – 1.04	0.462
Pcor × Group [BP]	1	0.92 – 1.08	0.951
Pcor × Group [MDD]	1.16	1.07 – 1.25	**<0.001**
(Set size × Neural set size marker) × Delay	1.01	0.94 – 1.07	0.857
(Set size × Neural set size marker) × Pcor	1.01	0.96 – 1.06	0.712
(Set size × Neural set size marker) × Group [SCZ]	1.01	0.93 – 1.10	0.832
(Set size × Neural set size marker) × Group [BP]	0.97	0.88 – 1.08	0.62
(Set size × Neural set size marker) × Group [MDD]	1.1	0.99 – 1.24	0.084
(Neural set size marker × Delay) × Group [SCZ]	0.96	0.88 – 1.05	0.422
(Neural set size marker × Delay) × Group [BP]	0.89	0.80 – 0.99	**0.039**
(Neural set size marker × Delay) × Group [MDD]	1.09	0.98 – 1.21	0.114
(Neural set size marker × Pcor) × Group [SCZ]	1.02	0.95 – 1.09	0.59
(Neural set size marker × Pcor) × Group [BP]	1.06	0.98 – 1.14	0.147
(Neural set size marker × Pcor) × Group [MDD]	0.95	0.88 – 1.03	0.234
(Delay × Pcor) × Group [SCZ]	1.06	0.98 – 1.15	0.141
(Delay × Pcor) × Group [BP]	1.03	0.94 – 1.13	0.536
(Delay × Pcor) × Group [MDD]	1.02	0.94 – 1.12	0.617

Table S9 continues.

Table S9 continued.

(Set size × Neural set size marker × Delay) × Group [SCZ]	1.1	1.01 – 1.20	**0.0`31**
(Set size × Neural set size marker × Delay) × Group [BP]	1.23	1.12 – 1.35	**<0.001**
(Set size × Neural set size marker × Delay) × Group [MDD]	0.98	0.89 – 1.09	0.727
(Set size × Neural set size marker × Pcor) × Group [SCZ]	1.07	1.00 – 1.15	0.052
(Set size × Neural set size marker × Pcor) × Group [BP]	0.99	0.92 – 1.08	0.879
(Set size × Neural set size marker × Pcor) × Group [MDD]	1.13	1.04 – 1.22	**0.004**
**Random Effects**			
σ^2	3.29		
$\tau_{00\ \text{sbj:Group}}$	0.89		
τ_{00} Group	0		
N $_{\text{sbj}}$	222		
N Group	4		
Observations	58381		
Marginal R^2	0.169		

Output from multi-linear mixed regression model fitted across all participants and including clinical group (SCZ = schizophrenia, BP = bipolar disorder, MDD = major depressive disorder) as an additional factor. The participants without a mental health disorder diagnosis (CTRL) served as reference group. Regression model included accuracy as binary dependent variable, corrects (1) and errors (0). Predictors included: clinical group, delay, set size, neural set size marker, and previous correct responses indexing reward history (pcor) as well as their interactions. Shown are means and 95% confidence intervals of fixed effects as well as estimated random effects.

Table S10. Regression output of clinical model with accuracy as dependent variable.

**Dependent variable: Accuracy**			
Predictors	*Odds Ratios*	*CI*	*p*
(Intercept)	19.36	16.04 – 23.37	**<0.001**
Set size	0.9	0.84 – 0.96	**0.001**
Delay	0.71	0.66 – 0.76	**<0.001**
Pcor	1.6	1.53 – 1.68	**<0.001**
Group [SCZ]	0.44	0.33 – 0.58	**<0.001**
Group [BP]	0.68	0.50 – 0.93	**0.016**
Group [MDD]	1.01	0.75 – 1.37	0.93
Set size × Delay	0.69	0.66 – 0.73	**<0.001**
Set size × Pcor	0.94	0.89 – 0.98	**0.008**
Delay × Pcor	1.24	1.17 – 1.32	**<0.001**
Set size × Group [SCZ]	0.93	0.85 – 1.01	0.077
Set size × Group [BP]	0.87	0.79 – 0.97	**0.01**
Set size × Group [MDD]	0.85	0.76 – 0.95	**0.003**
Delay × Group [SCZ]	0.93	0.85 – 1.03	0.146
Delay × Group [BP]	0.91	0.81 – 1.02	0.108
Delay × Group [MDD]	1.11	0.99 – 1.24	0.08
Pcor × Group [SCZ]	0.99	0.93 – 1.05	0.742
Pcor × Group [BP]	0.99	0.92 – 1.06	0.697
Pcor × Group [MDD]	1.11	1.03 – 1.19	**0.005**
(Set size × Delay) × Group [SCZ]	1.04	0.97 – 1.11	0.262
(Set size × Delay) × Group [BP]	1.02	0.94 – 1.11	0.633
(Set size × Delay) × Group [MDD]	1.01	0.93 – 1.10	0.76
(Set size × Pcor) × Group [SCZ]	1.08	1.00 – 1.15	**0.039**
(Set size × Pcor) × Group [BP]	1.04	0.95 – 1.13	0.379
(Set size × Pcor) × Group [MDD]	1	0.92 – 1.08	0.951
(Delay × Pcor) × Group [SCZ]	0.99	0.91 – 1.07	0.761
(Delay × Pcor) × Group [BP]	1	0.90 – 1.10	0.958
(Delay × Pcor) × Group [MDD]	1	0.91 – 1.09	0.961
**Random Effects**			
σ^2	3.29		
$\tau_{00\ sbj:Group}$	0.69		
τ_{00} Group	0		
N_{sbj}	255		
N Group	4		
Observations	68475		
Marginal R^2	0.179		

Output from multi-linear mixed regression model fitted across all participants and including clinical group (SCZ = schizophrenia, BP = bipolar disorder, MDD = major depressive disorder) as an additional factor. The participants without a mental health disorder diagnosis (CTRL) served as reference group. Regression

model included accuracy as binary dependent variable, corrects (1) and errors (0). Predictors included: clinical group, delay, set size, and previous correct responses indexing reward history (pcor) as well as their interactions. Shown are means and 95% confidence intervals of fixed effects as well as estimated random effects.

Table S11. Regression output of clinical model with EEG-SetSize as dependent variable.

**Dependent variable: EEG-SetSize**			
Predictors	*Estimates*	*CI*	*p*
(Intercept)	0.03	0.01 – 0.04	**<0.001**
Set size	0.12	0.11 – 0.14	**<0.001**
Group [SCZ]	0	-0.03 – 0.02	0.711
Group [BP]	0	-0.02 – 0.02	0.913
Group [MDD]	-0.01	-0.03 – 0.01	0.408
Set size × Pcor	0.01	-0.00 – 0.03	0.093
Set size × Group [SCZ]	-0.04	-0.06 – -0.02	**0.001**
Set size × Group [BP]	-0.03	-0.05 – -0.01	**0.016**
Set size × Group [MDD]	-0.01	-0.03 – 0.02	0.62
Set size × Pcor × Group [SCZ]	0.01	-0.02 – 0.03	0.622
Set size × Pcor × Group [BP]	0	-0.03 – 0.02	0.822
Set size × Pcor × Group [MDD]	-0.01	-0.04 – 0.01	0.232
**Random Effects**			
σ^2	0.98		
$\tau_{00\ sbj}$	0		
N_{sbj}	222		
Observations	58381		
Marginal R^2	0.012		

Output from multi-linear mixed regression model fitted across all participants and including clinical group (SCZ = schizophrenia, BP = bipolar disorder, MDD = major depressive disorder) as an additional factor. The participants without a mental health disorder diagnosis (CTRL) served as reference group. Regression model included neural set size marker as dependent variable. Predictors included: clinical group, set size, and its interaction with previous correct responses indexing reward history (pcor). Shown are means and 95% confidence intervals of fixed effects as well as estimated random effects.

Table S12. Regression output of clinical model with EEG-Q as dependent variable.

**Dependent variable: EEG-Q**			
Predictors	*Estimates*	*CI*	*p*
(Intercept)	0	-0.02 – 0.02	0.816
Pcor	0.13	0.11 – 0.14	**<0.001**
Neural set size marker	-0.31	-0.33 – -0.30	**<0.001**
Set size	0.07	0.05 – 0.09	**<0.001**
Group [SCZ]	0	-0.03 – 0.03	0.964
Group [BP]	0	-0.03 – 0.03	0.889
Group [MDD]	0.01	-0.02 – 0.04	0.667
Pcor × Neural set size marker	0.02	0.00 – 0.03	**0.02**
Pcor × Set size	0	-0.01 – 0.02	0.581
Neural set size marker × Set size	0	-0.01 – 0.02	0.861
Pcor × Group [SCZ]	-0.02	-0.05 – 0.00	0.052
Pcor × Group [BP]	-0.01	-0.04 – 0.01	0.395
Pcor × Group [MDD]	0	-0.02 – 0.02	0.937
Neural set size marker × Group [SCZ]	-0.03	-0.05 – -0.01	**0.005**
Neural set size marker × Group [BP]	-0.03	-0.06 – -0.01	**0.004**
Neural set size marker × Group [MDD]	-0.02	-0.04 – 0.00	0.086
Set size × Group [SCZ]	-0.01	-0.04 – 0.03	0.673
Set size × Group [BP]	-0.05	-0.08 – -0.01	**0.014**
Set size × Group [MDD]	-0.01	-0.05 – 0.02	0.485
(Pcor × Neural set size marker) × Set size	-0.01	-0.02 – 0.01	0.212
(Pcor × Neural set size marker) × Set size × Group [SCZ]	-0.02	-0.04 – 0.00	0.104
(Pcor × Neural set size marker) × Set size × Group [BP]	0	-0.02 – 0.02	0.931
(Pcor × Neural set size marker) × Set size × Group [MDD]	-0.01	-0.03 – 0.01	0.424
(Pcor × Set size) × Group [SCZ]	-0.01	-0.03 – 0.02	0.489
(Pcor × Set size) × Group [BP]	0	-0.03 – 0.02	0.835
(Pcor × Set size) × Group [MDD]	0.01	-0.01 – 0.04	0.299
(Neural set size marker × Set size) × group [SCZ]	0	-0.03 – 0.02	0.732
(Neural set size marker × Set size) × group [BP]	0	-0.03 – 0.02	0.721
(Neural set size marker × Set size) × group [MDD]	-0.01	-0.04 – 0.01	0.255
(Pcor × neural set size marker × Set size) × Group [SCZ]	0.01	-0.01 – 0.03	0.366
(Pcor × neural set size marker × Set size) × Group [BP]	0	-0.03 – 0.02	0.846
(Pcor × neural set size marker × Set size) × Group [MDD]	0.01	-0.01 – 0.03	0.36
**Random Effects**			
σ^2	0.86		
$\tau_{00\ SetSize}$	0.01		
$\tau_{00\ sbj}$	0		
$\tau_{11\ SetSize.Pcor}$	0		
$\tau_{11\ sbj.SetSize}$	0		

Table S12 continues.

Table S12 continued.

$\rho_{01\ SetSize}$	0.22
$\rho_{01\ sbj}$	0
N_{sbj}	222
$N_{SetSize}$	888
Observations	51794
Marginal R^2	0.124

Output from multi-linear mixed regression model fitted across all participants and including clinical group (SCZ = schizophrenia, BP = bipolar disorder, MDD = major depressive disorder) as an additional factor. The participants without a mental health disorder diagnosis (CTRL) served as reference group. Regression model included neural Q marker as dependent variable. Predictors included: clinical group, set size, neural set size marker, previous correct responses indexing reward history (pcor), and their interactions. Shown are means and 95% confidence intervals of fixed effects as well as estimated random effects.

Table S13. Regression output of clinical model with EEG-SetSize as dependent variable.

**Dependent variable: EEG-SetSize**			
Predictors	*Estimates*	*CI*	*p*
(Intercept)	0.03	0.01 – 0.04	**<0.001**
Set size	0.15	0.13 – 0.16	**<0.001**
Delay	-0.03	-0.05 – -0.02	**<0.001**
Pcor	0.03	0.01 – 0.04	**<0.001**
Group [SCZ]	-0.01	-0.03 – 0.02	0.673
Group [BP]	0	-0.03 – 0.02	0.756
Group [MDD]	-0.01	-0.04 – 0.01	0.268
Set size × Delay	0.02	0.00 – 0.03	**0.049**
Set size × Pcor	0	-0.01 – 0.02	0.583
Delay × Pcor	0.01	-0.01 – 0.02	0.251
Set size × Group [SCZ]	-0.03	-0.05 – -0.00	**0.021**
Set size × Group [BP]	-0.02	-0.04 – 0.01	0.192
Set size × Group [MDD]	0	-0.03 – 0.02	0.722
Delay × Group [SCZ]	-0.02	-0.04 – 0.01	0.202
Delay × Group [BP]	-0.03	-0.05 – -0.00	**0.039**
Delay × Group [MDD]	0	-0.03 – 0.02	0.693
Pcor × Group [SCZ]	0	-0.03 – 0.02	0.681
Pcor × Group [BP]	0	-0.02 – 0.03	0.838
Pcor × Group [MDD]	-0.03	-0.05 – -0.00	**0.032**
(Set size × Delay) × Group [SCZ]	0.01	-0.01 – 0.04	0.302
(Set size × Delay) × Group [BP]	0.01	-0.01 – 0.04	0.295
(Set size × Delay) × Group [MDD]	0	-0.02 – 0.03	0.698
(Set size × Pcor) × Group [SCZ]	0.02	-0.01 – 0.04	0.149
(Set size × Pcor) × Group [BP]	0	-0.02 – 0.03	0.819
(Set size × Pcor) × Group [MDD]	0	-0.02 – 0.02	0.955
(Delay × Pcor) × Group [SCZ]	0	-0.03 – 0.02	0.822
(Delay × Pcor) × Group [BP]	0	-0.03 – 0.02	0.698
(Delay × Pcor) × Group [MDD]	0	-0.03 – 0.02	0.761
**Random Effects**			
σ^2	0.97		
$\tau_{00\ sbj}$	0		
N_{sbj}	222		
Observations	51794		
Marginal R^2	0.017		

Output from multi-linear mixed regression model fitted across all participants and including clinical group (SCZ = schizophrenia, BP = bipolar disorder, MDD = major depressive disorder) as an additional factor. The participants without a mental health disorder diagnosis (CTRL) served as reference group. Regression model included neural set size marker as dependent variable. Predictors included: clinical group, set size,

delay, previous correct responses indexing reward history (pcor), and their two-way interactions with group. Shown are means and 95% confidence intervals of fixed effects as well as estimated random effects.

Table S14. Regression output of clinical model with EEG-SetSize as dependent variable.

**Dependent variable: EEG-SetSize**			
Predictors	*Estimates*	*CI*	*p*
(Intercept)	0.04	0.02 – 0.05	**<0.001**
Set size	0.13	0.11 – 0.15	**<0.001**
Group [SCZ]	0	-0.02 – 0.02	0.989
Group [BP]	0	-0.02 – 0.03	0.849
Group [MDD]	-0.01	-0.04 – 0.01	0.305
Set size × Group [SCZ]	-0.04	-0.07 – -0.01	**0.014**
Set size × Group [BP]	-0.04	-0.07 – -0.00	**0.037**
Set size × Group [MDD]	-0.01	-0.04 – 0.02	0.661
**Random Effects**			
σ^2	0.97		
$\tau_{00\ sbj}$	0		
$\tau_{11\ sbj.SetSize}$	0		
$\rho_{01\ sbj}$	-1		
N $_{sbj}$	222		
Observations	51794		
Marginal R^2	0.014		

Output from multi-linear mixed regression model fitted across all participants and including clinical group (SCZ = schizophrenia, BP = bipolar disorder, MDD = major depressive disorder) as an additional factor. The participants without a mental health disorder diagnosis (CTRL) served as reference group. Regression model included neural set size marker as dependent variable. Predictors included: clinical group, set size and their interactions. Shown are means and 95% confidence intervals of fixed effects as well as estimated random effects.

Table S15. Regression output of clinical model with EEG-RPE as dependent variable.

**Dependent variable: EEG-RPE**			
Predictors	*Estimates*	*CI*	*p*
(Intercept)	-0.16	-0.19 – -0.14	**<0.001**
Set size	0.06	0.04 – 0.08	**<0.001**
Neural set size marker	0.02	-0.00 – 0.03	0.105
Group [SCZ]	0.05	0.02 – 0.09	**0.002**
Group [BP]	0.05	0.01 – 0.09	**0.01**
Group [MDD]	-0.01	-0.05 – 0.03	0.629
Set size × neural set size marker	0	-0.02 – 0.01	0.831
Set size × Group [SCZ]	0.02	-0.01 – 0.05	0.267
Set size × Group [BP]	0.02	-0.02 – 0.06	0.252
Set size × Group [MDD]	0	-0.04 – 0.03	0.937
Neural set size marker × Group [SCZ]	-0.05	-0.07 – -0.02	**0.001**
Neural set size marker × Group [BP]	-0.04	-0.08 – -0.01	**0.005**
Neural set size marker × Group [MDD]	-0.02	-0.05 – 0.00	**0.049**
(Set size × Neural set size marker) × Group [SCZ]	-0.03	-0.05 – -0.01	**0.014**
(Set size × Neural set size marker) × Group [BP]	0	-0.03 – 0.02	0.911
(Set size × Neural set size marker) × Group [MDD]	0.01	-0.01 – 0.04	0.287
**Random Effects**			
σ^2	0.95		
$\tau_{00\ \mathrm{sbj}}$	0.01		
$\tau_{11\ \mathrm{sbj.SetSize}}$	0.01		
$\tau_{11\ \mathrm{sbj.NeuralSetSizeMarker}}$	0		
$\tau_{11\ \mathrm{sbj.SetSize:NeuralSetSizeMarker}}$	0		
ρ_{01}	0.04		
	0		
	0.03		
N $_{\mathrm{sbj}}$	222		
Observations	51794		
Marginal R^2	0.006		

Output from multi-linear mixed regression model fitted across all participants and including clinical group (SCZ = schizophrenia, BP = bipolar disorder, MDD = major depressive disorder) as an additional factor. The participants without a mental health disorder diagnosis (CTRL) served as reference group. Regression model included neural reward prediction error (RPE) as dependent variable. Predictors included: clinical group, set size, neural set size marker, and their interactions. Shown are means and 95% confidence intervals of fixed effects as well as estimated random effects.

Table S16. Regression output of clinical model with EEG-RPE as dependent variable.

**Dependent variable: EEG-RPE**			
Predictors	*Estimates*	*CI*	*p*
(Intercept)	-0.18	-0.20 – -0.15	**<0.001**
Set size	0.05	0.04 – 0.07	**<0.001**
Delay	0.03	0.02 – 0.05	**<0.001**
Pcor	-0.16	-0.17 – -0.14	**<0.001**
Group [SCZ]	0.04	0.01 – 0.08	**0.009**
Group [BP]	0.04	0.00 – 0.07	**0.048**
Group [MDD]	-0.01	-0.05 – 0.02	0.5
Set size × Delay	0.02	0.01 – 0.04	**0.001**
Set size × Pcor	-0.02	-0.03 – -0.00	**0.018**
Delay × Pcor	-0.05	-0.06 – -0.03	**<0.001**
Set size × Group [SCZ]	0.02	-0.00 – 0.04	0.102
Set size × Group [BP]	0.03	-0.00 – 0.05	0.058
Set size × Group [MDD]	0	-0.02 – 0.03	0.86
Delay × Group [SCZ]	0	-0.03 – 0.02	0.782
Delay × Group [BP]	0	-0.03 – 0.02	0.983
Delay × Group [MDD]	0	-0.02 – 0.02	0.985
Pcor × Group [SCZ]	0	-0.02 – 0.02	0.939
Pcor × Group [BP]	-0.03	-0.05 – -0.00	**0.022**
Pcor × Group [MDD]	-0.03	-0.05 – -0.01	**0.006**
(Set size × Delay) × Group [SCZ]	0.01	-0.01 – 0.03	0.447
(Set size × Delay) × Group [BP]	0.03	0.00 – 0.05	**0.036**
(Set size × Delay) × Group [MDD]	0.01	-0.01 – 0.04	0.265
(Set size× Pcor) × Group [SCZ]	-0.01	-0.03 – 0.02	0.537
(Set size× Pcor) × Group [BP]	0.01	-0.01 – 0.04	0.354
(Set size× Pcor) × Group [MDD]	-0.01	-0.04 – 0.01	0.293
(Delay × Pcor) × Group [SCZ]	0.01	-0.01 – 0.03	0.362
(Delay × Pcor) × Group [BP]	0	-0.02 – 0.03	0.769
(Delay × Pcor) × Group [MDD]	-0.02	-0.04 – 0.00	0.053
**Random Effects**			
σ^2	0.93		
$\tau_{00\ sbj}$	0		
N_{sbj}	222		
Observations	51794		
Marginal R^2	0.041		

Output from multi-linear mixed regression model fitted across all participants and including clinical group (SCZ = schizophrenia, BP = bipolar disorder, MDD = major depressive disorder) as an additional factor. The participants without a mental health disorder diagnosis (CTRL) served as reference group. Regression model included neural reward prediction error (RPE) as dependent variable. Predictors included: clinical group, set size, delay, previous correct responses indexing reward history (pcor), and their two-way

interactions with group. Shown are means and 95% confidence intervals of fixed effects as well as estimated random effects.

Table S17. Regression outputs of sensitivity analyses showing dependency of model-based EEG measures by study site.

A

	EEG-RPE		
Predictors	*Estimates*	*std. Error*	*p*
(Intercept)	-0.13	0.02	**<0.001**
site	-0.00	0.00	0.301
**Random Effects**			
σ^2	0.97		
$\tau_{00\ src\_subject\_id}$	0.01		
ICC	0.01		
$N_{src\_subject\_id}$	221		
Observations	51634		
Marginal R^2 / Conditional R^2	0.000 / 0.006		

B

	EEG-Q		
Predictors	*Estimates*	*std. Error*	*p*
(Intercept)	0.03	0.01	**0.002**
site	0.01	0.00	**0.015**
**Random Effects**			
σ^2	0.99		
$\tau_{00\ src\_subject\_id}$	0.00		
$N_{src\_subject\_id}$	221		
Observations	51634		
Marginal R^2 / Conditional R^2	0.000 / NA		

C

	EEG-SetSize		
Predictors	*Estimates*	*std. Error*	*p*
(Intercept)	0.02	0.01	**0.022**
site	0.00	0.00	0.385
**Random Effects**			
σ^2	0.99		
$\tau_{00\ src\_subject\_id}$	0.00		
$N_{src\_subject\_id}$	221		
Observations	51634		
Marginal R^2 / Conditional R^2	0.000 / NA		

Output from multi-linear mixed regression model fitted across all participants including study site as independent variable and the main EEG measures extracted from the model-based analyses as dependent variables. In sum, site had no significant effect on EEG-RPE or EEG-SetSize. A weak effect was observed for EEG-Q (B = 0.01, SE = 0.00), but this effect was small and unlikely to be of practical significance. **A.** Regression model included neural reward prediction error (RPE) as dependent variable. **B.** Regression model included neural Q marker as dependent variable. **C.** Regression model included neural set size marker as dependent variable. Shown are means and 95% confidence intervals of fixed effects as well as estimated random effects.

Table S18. Regression outputs of sensitivity analyses showing dependency of model-based EEG measures by study site and group.

A

	EEG-RPE		
Predictors	*Estimates*	*std. Error*	*p*
(Intercept)	-0.17	0.03	**<0.001**
site	0.00	0.01	0.875
Group [SCZ]	0.09	0.04	**0.011**
Group [Bip]	0.04	0.05	0.462
Group [Dep]	0.01	0.04	0.722
site × Group [SCZ]	-0.02	0.01	0.133
site × Group [Bip]	0.00	0.01	0.826
site × Group [Dep]	-0.01	0.01	0.551
**Random Effects**			
σ^2	0.97		
$\tau_{00\ src\_subject\_id}$	0.01		
ICC	0.01		
$N_{src\_subject\_id}$	221		
Observations	51634		
Marginal R^2 / Conditional R^2	0.001 / 0.007		

B

	EEG-Q		
Predictors	*Estimates*	*std. Error*	*p*
(Intercept)	0.03	0.02	**0.040**
site	0.01	0.00	0.131
Group [SCZ]	-0.00	0.02	0.983
Group [Bip]	-0.02	0.03	0.538
Group [Dep]	-0.00	0.03	0.902
site × Group [SCZ]	-0.00	0.01	0.791
site × Group [Bip]	0.00	0.01	0.891
site × Group [Dep]	0.00	0.01	0.997
**Random Effects**			
σ^2	0.99		
$\tau_{00\ src\_subject\_id}$	0.00		
$N_{src\_subject\_id}$	221		
Observations	51634		
Marginal R^2 / Conditional R^2	0.000 / NA		

C

	EEG-SetSize		
Predictors	*Estimates*	*std. Error*	*p*
(Intercept)	0.01	0.02	0.399
site	0.01	0.00	0.191
Group [SCZ]	0.03	0.02	0.275
Group [Bip]	0.03	0.03	0.274
Group [Dep]	-0.02	0.03	0.457
site × Group [SCZ]	-0.01	0.01	0.181
site × Group [Bip]	-0.01	0.01	0.221
site × Group [Dep]	0.00	0.01	0.803
**Random Effects**			
σ^2	0.99		
$\tau_{00\ src\_subject\_id}$	0.00		
$N_{src\_subject\_id}$	221		
Observations	51634		
Marginal R^2 / Conditional R^2	0.000 / NA		

Output from multi-linear mixed regression model fitted across all participants including the main EEG measures extracted from the model-based analyses as dependent variables and study site and clinical groups (SCZ = schizophrenia, BP = bipolar disorder, MDD = major depressive disorder) as additional factors. In sum, there were no group x site interactions, suggesting that our main findings were robust across study locations. **A.** Regression model included neural reward prediction error (RPE) as dependent variable. **B.** Regression model included neural Q marker as dependent variable. **C.** Regression model included neural set size marker as dependent variable. Shown are means and 95% confidence intervals of fixed effects as well as estimated random effects.

4. References

1. First MB, Williams JB, Karg RS, Spitzer RL. Structured clinical interview for DSM-5 disorders. Clinician Version (SCID-5-CV). Published online 2015.

2. Collins AGE, Frank MJ. How much of reinforcement learning is working memory, not reinforcement learning? A behavioral, computational, and neurogenetic analysis. *Eur J Neurosci*. 2012;35(7):1024-1035. doi:10.1111/j.1460-9568.2011.07980.x

3. Collins AGE, Ciullo B, Frank MJ, Badre D. Working Memory Load Strengthens Reward Prediction Errors. *J Neurosci*. 2017;37(16):4332-4342. doi:10.1523/JNEUROSCI.2700-16.2017

4. Collins AGE, Frank MJ. Within- and across-trial dynamics of human EEG reveal cooperative interplay between reinforcement learning and working memory. *Proc Natl Acad Sci*. 2018;115(10):2502-2507. doi:10.1073/pnas.1720963115

5. Rac-Lubashevsky R, Cremer A, Collins AGE, Frank MJ, Schwabe L. Neural Index of Reinforcement Learning Predicts Improved Stimulus–Response Retention under High Working Memory Load. *J Neurosci*. 2023;43(17):3131-3143. doi:10.1523/JNEUROSCI.1274-22.2023

6. Collins AGE, Albrecht MA, Waltz JA, Gold JM, Frank MJ. Interactions Among Working Memory, Reinforcement Learning, and Effort in Value-Based Choice: A New Paradigm and Selective Deficits in Schizophrenia. *Biol Psychiatry*. 2017;82(6):431-439. doi:10.1016/j.biopsych.2017.05.017

7. Boudewyn MA, Erickson MA, Winsler K, et al. Managing EEG studies: How to prepare and what to do once data collection has begun. *Psychophysiology*. 2023;60(11):e14365. doi:10.1111/psyp.14365
